# Supplementary material for: H3K27M induces defective chromatin spread of PRC2-mediated repressive H3K27me2/me3 and is essential for glioma tumorigenesis
Source: Nat Commun. 2019 Mar 19;10:1262. doi: 10.1038/s41467-019-09140-x (PMC6425035; doi:10.1038/s41467-019-09140-x)
Supplement: Supplementary file 1 — Supplementary Information [file 41467_2019_9140_MOESM1_ESM.pdf]

H3K27M induces defective chromatin spread of PRC2-mediated repressive H3K27me2/me3 and is essential for glioma tumorigenesis

Supplementary information

Harutyunyan *et al.*

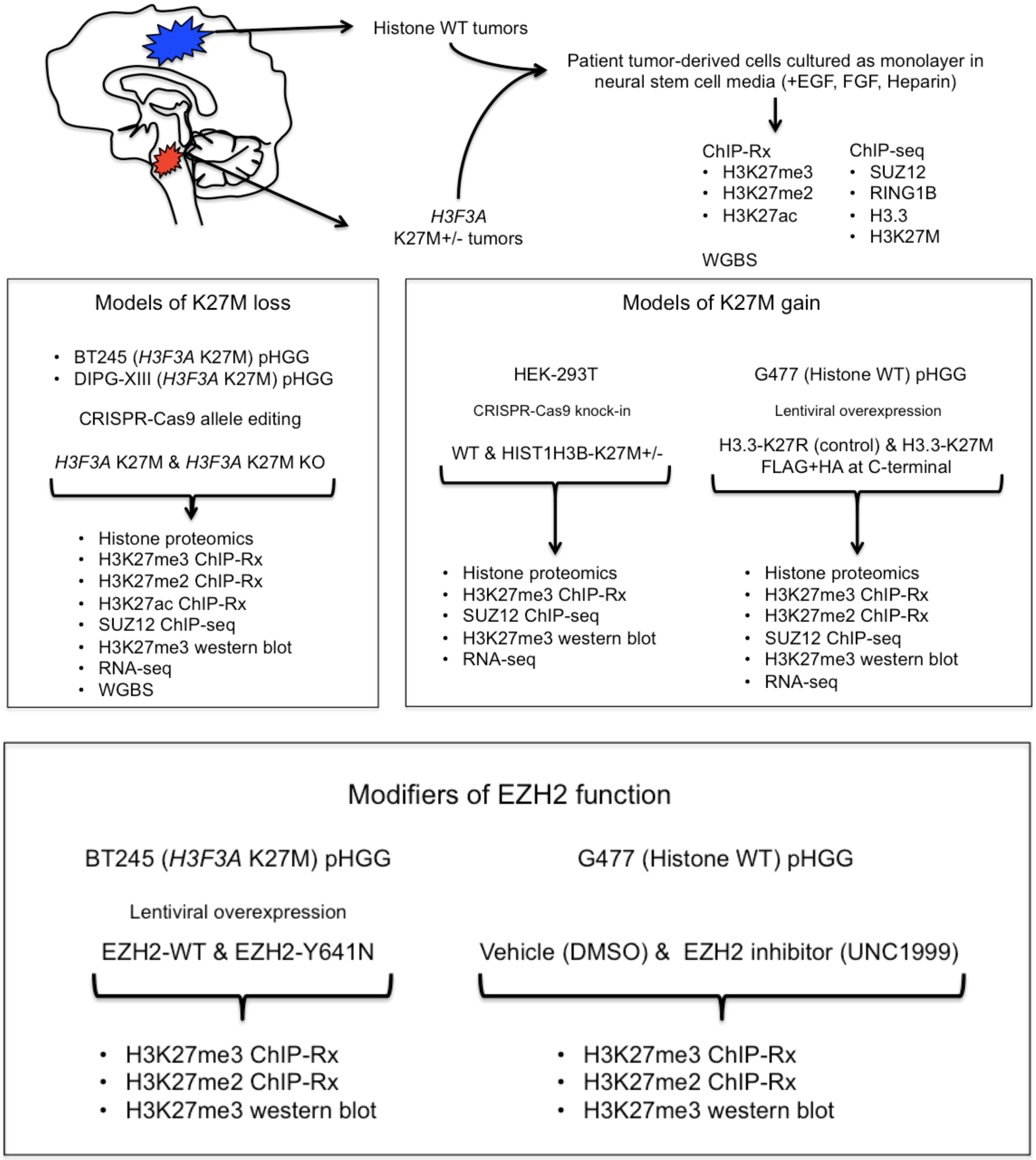

Supplementary Figure 1. Scheme of experimental datasets. Please note the color code used throughout the figures for cells: **red** is for cell lines expressing H3K27M mutation (primary pediatric high-grade gliomas (pHGG) lines, gene-edited cell lines, cells overexpressing H3K27M) and **blue** for cell lines wild-type (WT) for this mutation.

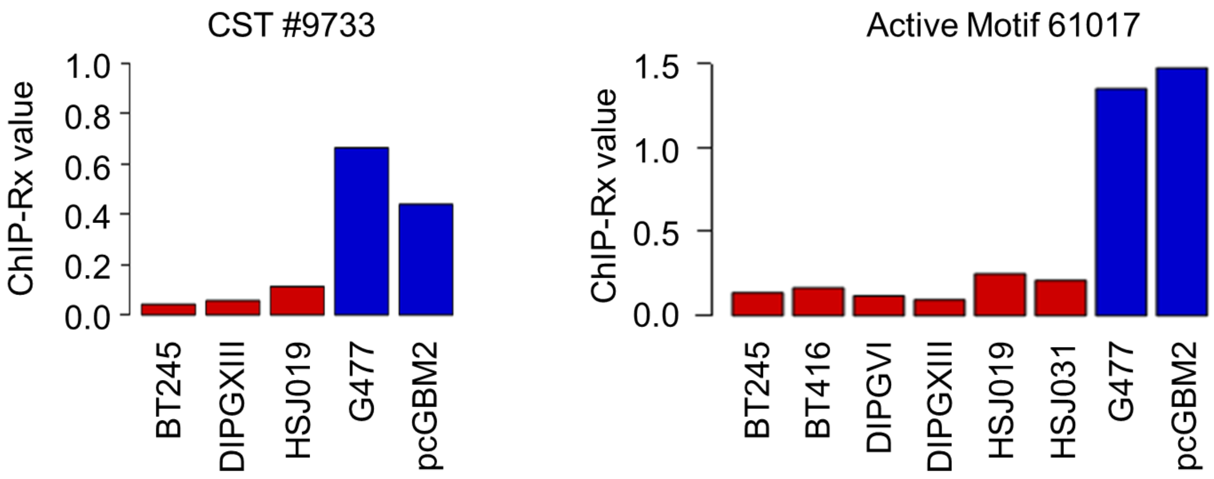

Supplementary Figure 2. ChIP-Rx values for H3K27me3 in different primary high-grade glioma (HGG) cell lines, using two different antibodies. Similar drastic decrease of the mark in H3.3K27M HGG cells (red) compared to wild-type HGG (blue) was observed using either anti-H3K27me3 antibody for ChIP-seq. Source data are provided as a Source Data file.

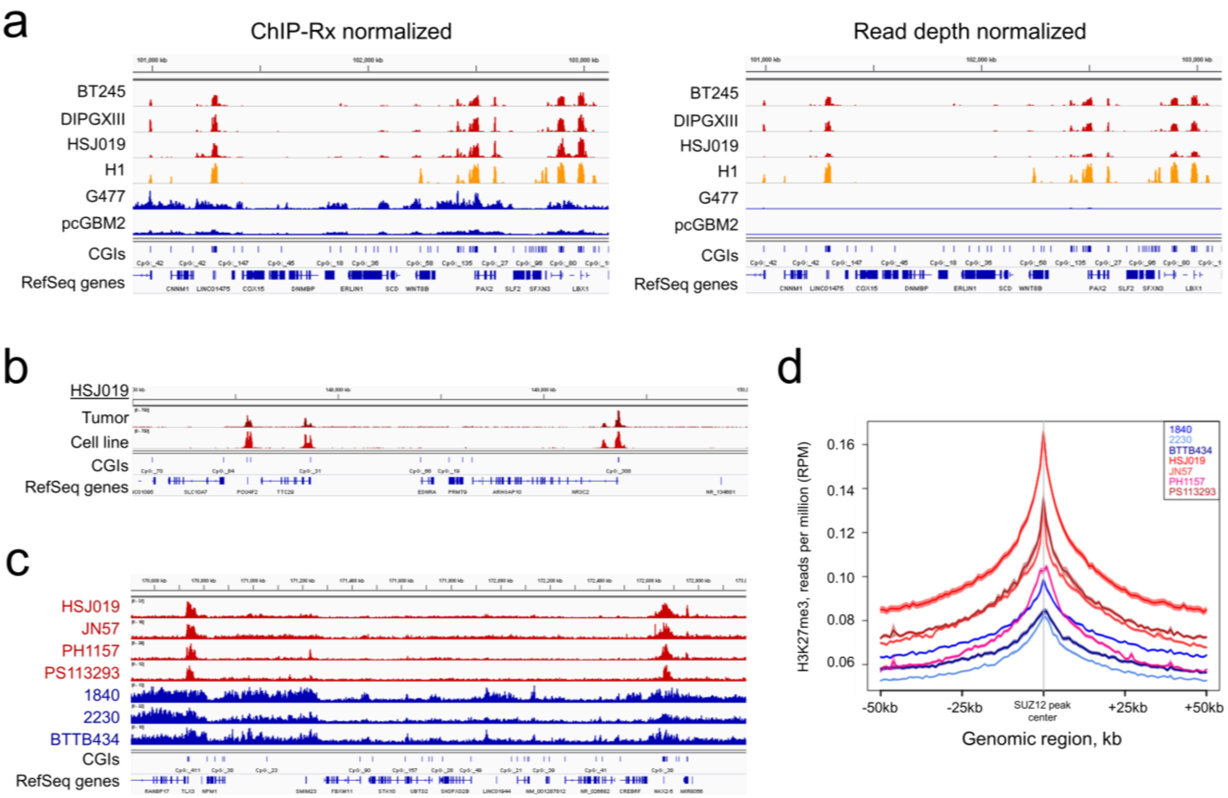

Supplementary Figure 3. a. Tracks of H3K27me3 for pediatric high-grade gliomas (pHGG)-derived primary cell lines and H1 human Embryonic Stem Cells (ESC), ChIP-Rx normalized (upper panel) and read depth normalized (lower panel). Note the significant differences that lead to overestimating the amount of H3K27me3 in H3K27M when using read depth (standard) normalization compared to ChIP-Rx. Note the “peaky” distribution of residual H3K27me3 in H3K27M cells (red tracks) which is centered around CpG islands (CGIs) like what is observed in H1 ESCs (orange track). This contrasts with the wide distribution of H3K27me3 in cells WT for this mutation (blue tracks). **b.** This distribution of residual H3K27me3 observed in primary cell lines mirrors the distribution of the mark in the original tumour as shown for the cell line where we had material for both (lower panel). Indeed, tracks of H3K27me3 for a tumor tissue of the primary sample HSJ019 and cell line derived from that tumor, ChIP-Rx normalized indicate that cell line models are reflective of the predominant epigenomic state of tumors *in vivo*. **c.** The distribution of residual H3K27me3 in primary tumors follows the same pattern as in cell lines when comparing H3.3 K27M (n=4) and H3 wild-type (n=3) tumor tissues. Tracks of H3K27me3 from glioblastoma tissue samples are shown, autoscaled due to the absence of drosophila spike-in. **d.** Aggregate plots of H3K27me3 in primary tumor tissue samples over common SUZ12 peaks derived from BT245 (H3.3K27M) and G477 (wild-type) cell lines. H3K27M mutant tumors are represented by shares of red, while wild-type tumors by shades of blue. Only in panels **c** and **d** we are using data derived from anti-H3K27me3 (Active Motif 61017) ChIP-seq.

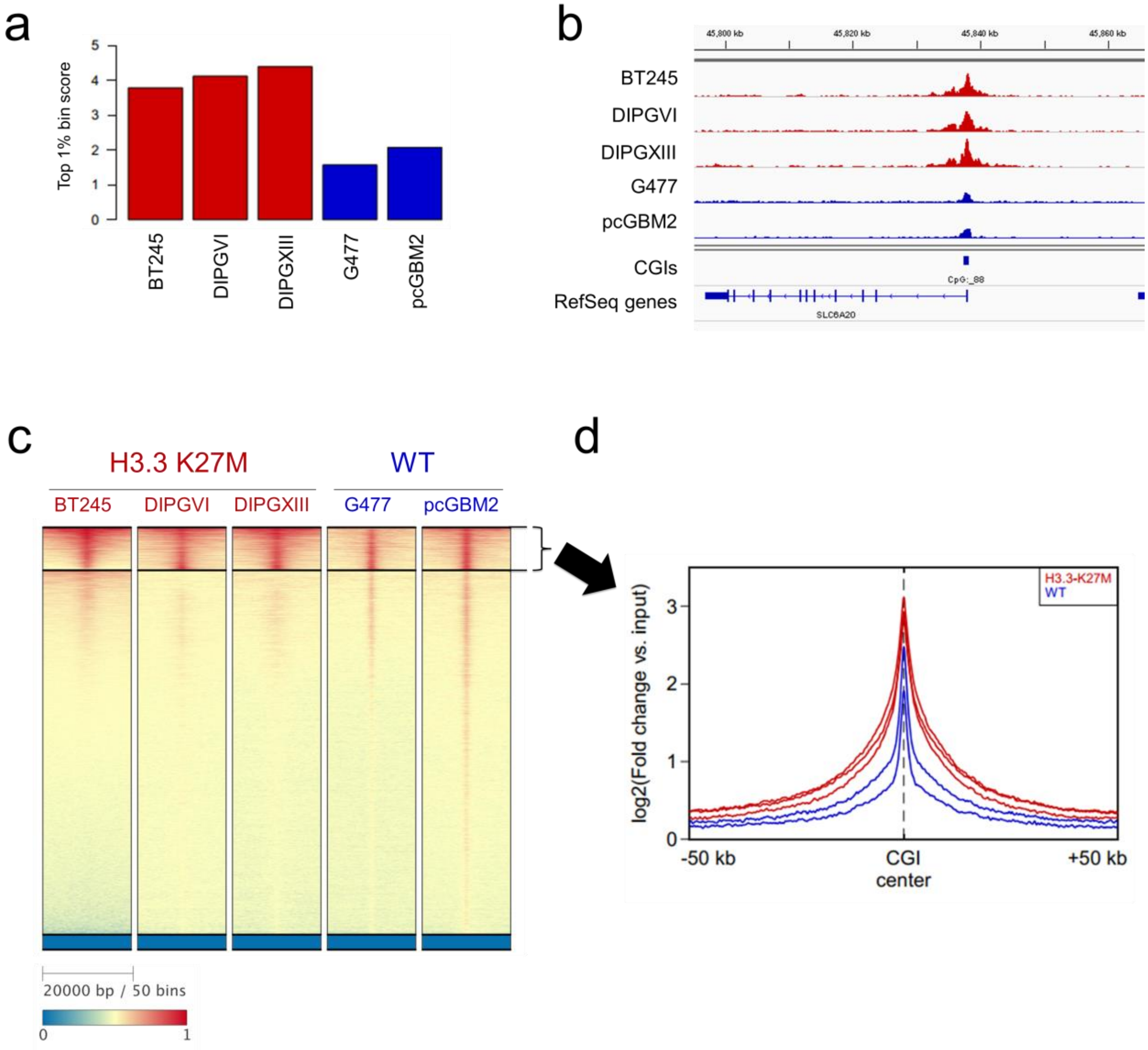

Supplementary Figure 4. SUZ12 deposition in H3K27M (red) is broader and enriched in unmethylated CGIs as compared to high-grade gliomas (HGG) lines wild-type (WT) for H3K27M (blue). **a.** SUZ12 top 1% 1kb bin scores and **b**. Representative SUZ12 ChIP-seq tracks, read depth normalized. **c.** heatmap plots at CGIs, primary cells, K27M vs. WT. Clustered by kmeans clustering (k=3). **d**. Aggregate plots of SUZ12 signal over CGIs for the top cluster. Source data are provided as a Source Data file.

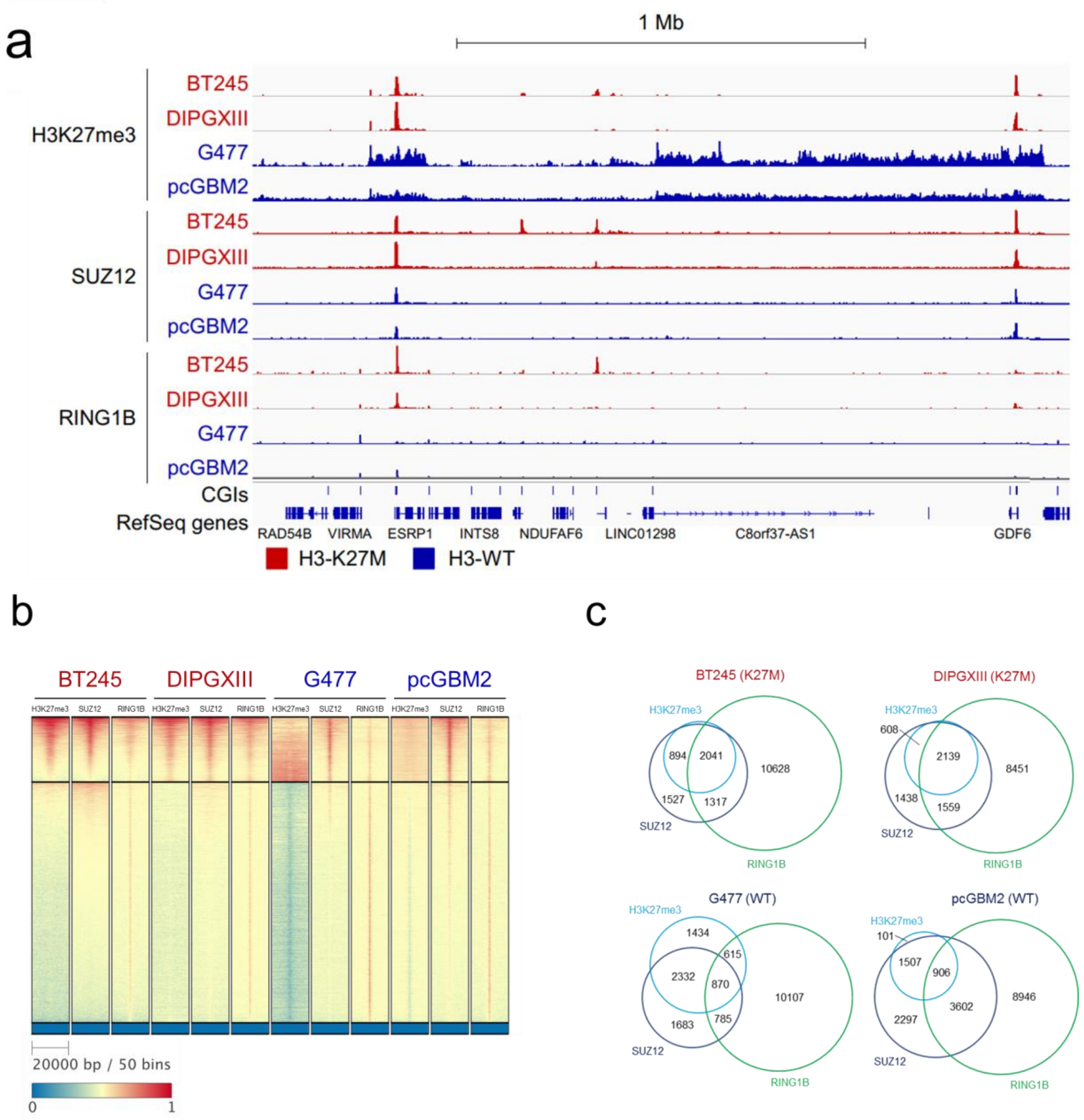

Supplementary Figure 5. a. Representative ChIP-seq tracks of H3K27me3, SUZ12 and RING1B for H3K27M and WT pHGG lines, showing differences in distribution of H3K27me3, SUZ12 and RING1B. **b.** Stacked heatmap plots for H3K27me3, SUZ12 and RING1B around CGIs showing stronger enrichment of RING1B in SUZ12/H3K27me3 positive regions in H3K27M cells. **c.** SUZ12/RING1B/H3K27me3 peaks overlap in wild-type (WT, G477, pcGBM2) and H3.3 K27M (BT245, DIPGXIII) HGG lines. H3K27me3 deposition strongly overlaps with SUZ12 sites in H3K27M lines BT245 and DIPGXIII compared to WT lines. Deposition of RING1B on these overlapping H3K27me3/SUZ12 sites shows ~ 2.5-fold enrichment in H3.3 K27M compared to WT.

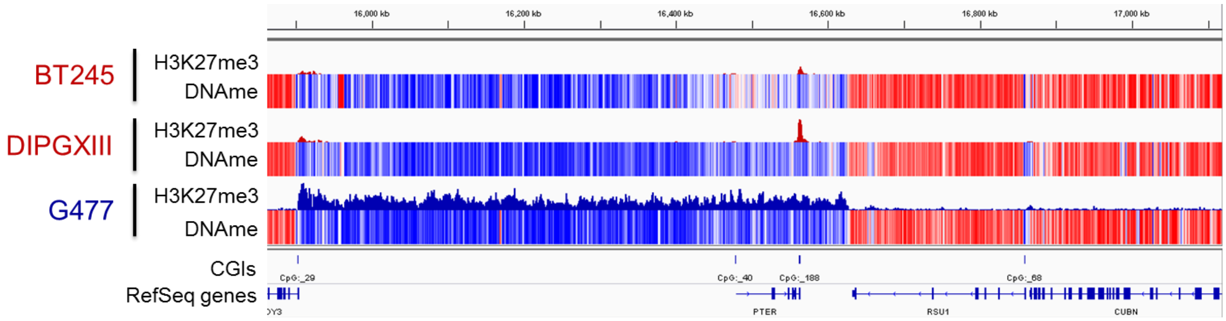

Supplementary Figure 6. Normalized ChIP-seq tracks demonstrating loss of H3K27me3 in partially methylated domain (PMD) regions in H3 K27M in pediatric HGG primary cells. DNA methylation is scaled from blue (0) to red (1) based on methylated base density calculated from whole genome-bisulfite sequencing.

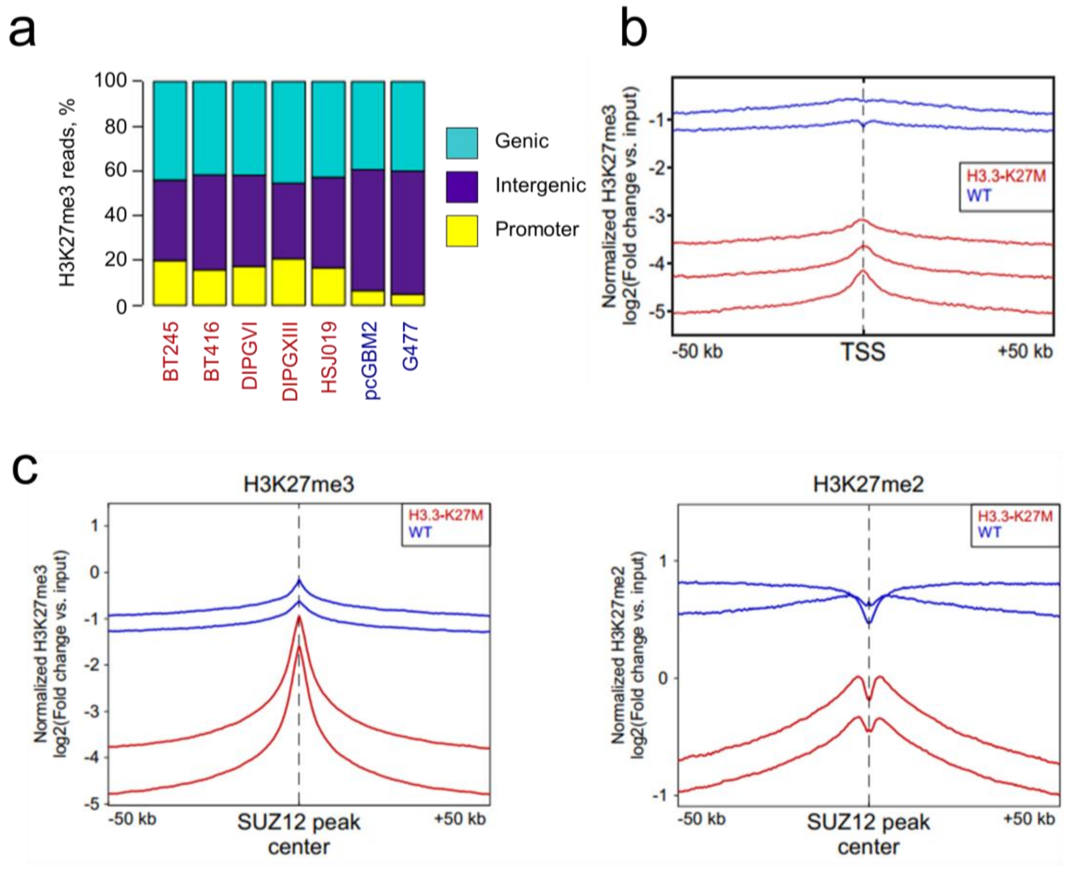

Supplementary Figure 7. H3K27me3 distribution in different genomic compartments. **a.** Promoter-genic-intergenic plot, primary cells. Proportion of H3K27me3 reads mapping to different genomic compartments (promoters, gene bodies or intergenic space) in wild-type or H3.3 K27M high-grade gliomas cell lines. **b.** H3K27me3 aggregate plots over TSS of commonly repressed genes in 5 analyzed cell lines (3 H3K27M, 2 WT). **c.** H3K27me2 and H3K27me3 aggregate plots over overlapping SUZ12 peaks in 4 analyzed cell lines (2 H3K27M, 2 WT). Source data are provided as a Source Data file.

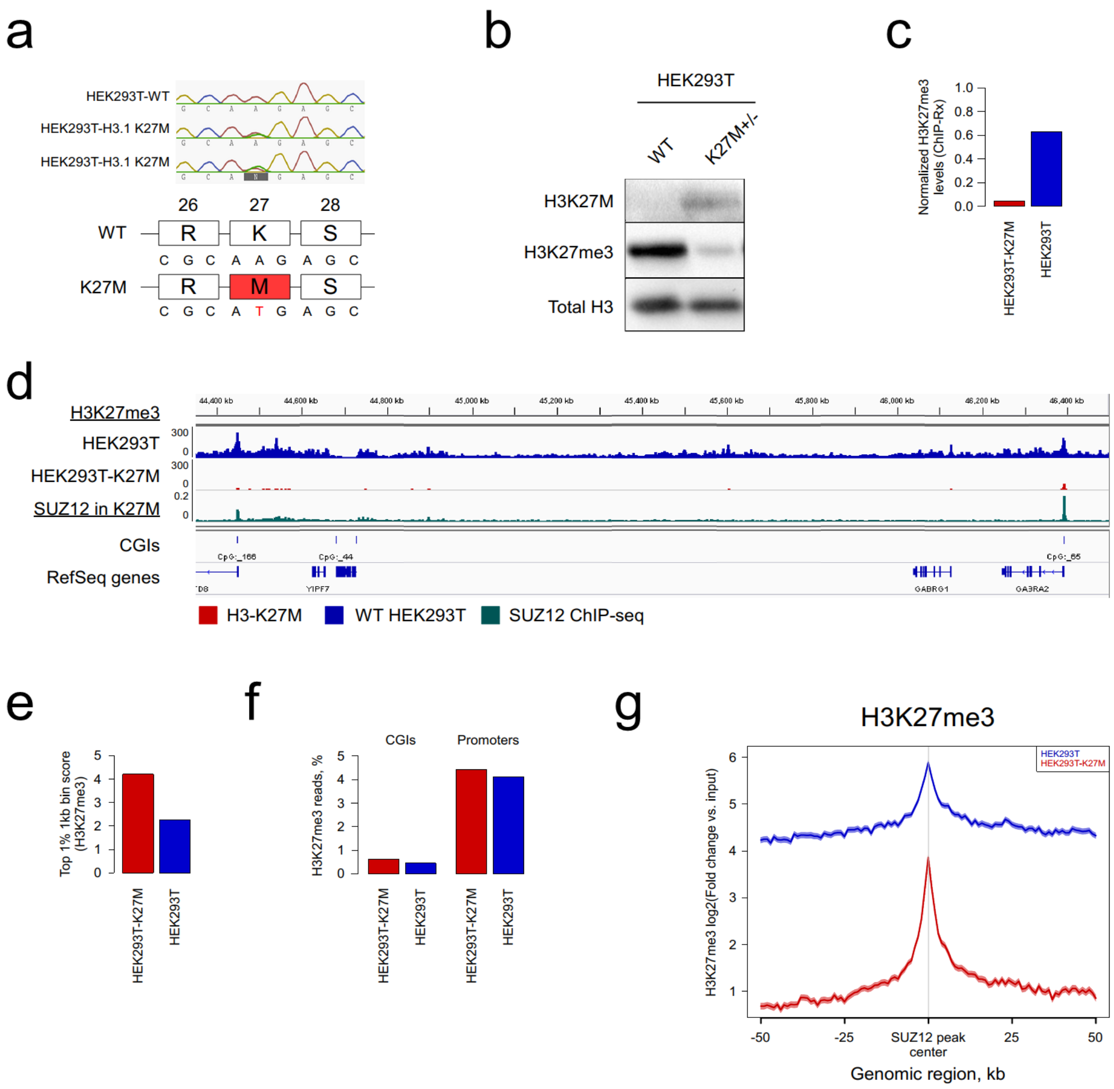

Supplementary Figure 8. Experimental introduction of H3.1K27M in HEK293T cell using CRISPR/Cas editing reproduces patterns of H3K27me3 distribution seen in H3K27M mutant high-grade gliomas. **a.** Sanger sequencing confirmation of CRISPR editing. Introduction of K27M induces a drastic decrease of H3K27me3 in HEK293T as shown using **b.** Western blot analysis or **c.** ChIP-Rx scores. Distribution of the H3K27me3 mark becomes confined to CGIs and mirrors H3K27M high -grade glioma lines as shown on **d.** ChIP-Rx normalized tracks, or using **e.** top 1% 1kb bin scores, **f.** proportion of H3K27me3 reads in CGIs or promoter regions and, **G.** SUZ12 peak centered aggregate plots of H3K27me3. Source data are provided as a Source Data file.

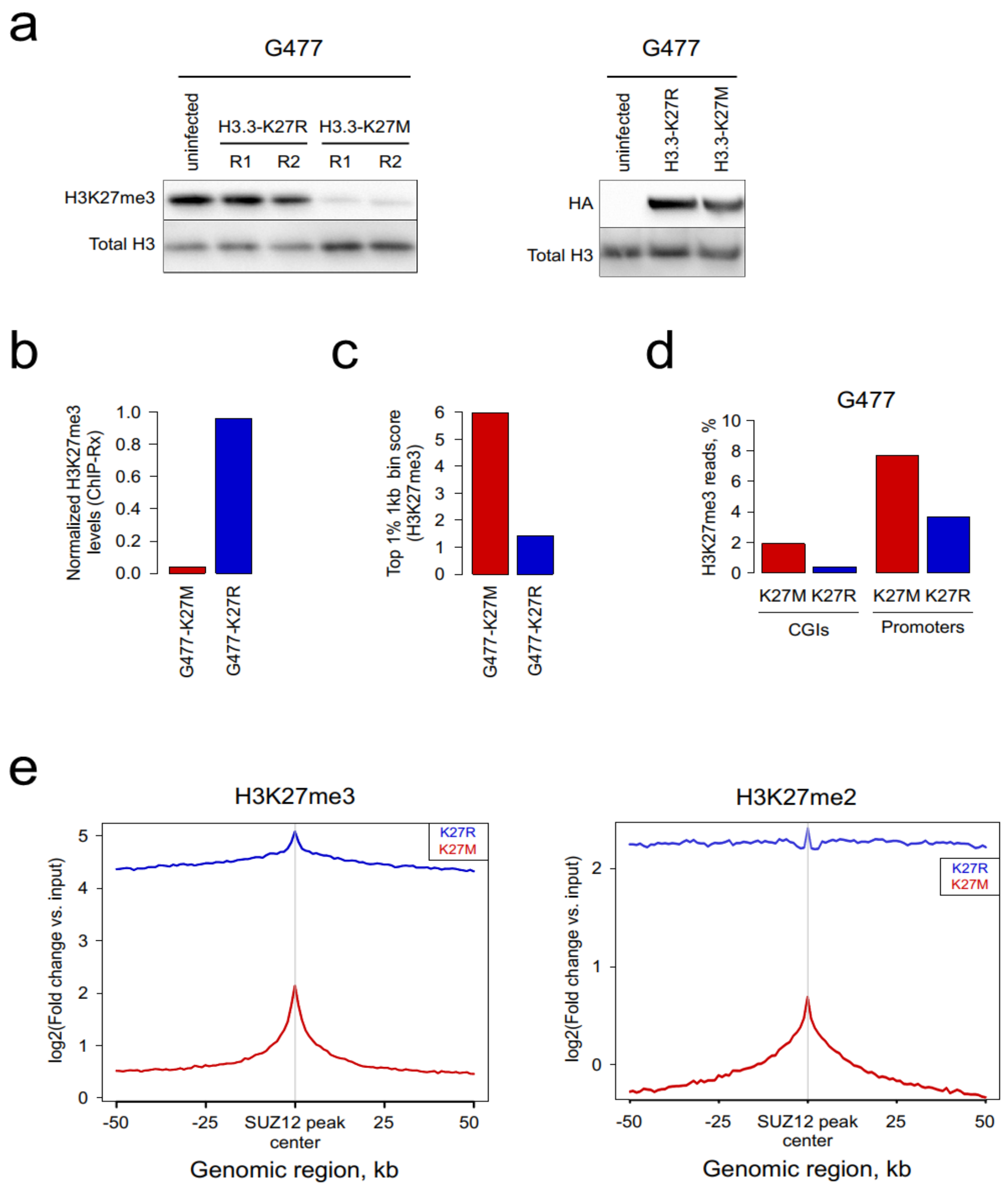

Supplementary Figure 9. H3K27me3 levels are drastically decreased in H3.3 K27M overexpressing G477 (red) compared to control G477 overexpressing the H3.3K27R mutation (blue), which was shown not to affect H3K27me3 levels[1]. **a.** Western blot showing decreased H3K27me3 levels in H3.3K27M expressing cells. Western blot of HA-tag confirms comparable levels of overexpression. **b.** ChIP-Rx scores and **c.** Top 1% 1kb bin scores further confirming decrease of the mark in H3.3K27M expressing G477cells. **d**. Proportion of H3K27me3 reads in CGIs or promoter regions and, **e.** SUZ12 peak centered aggregate plots of H3K27me3 and H3K27me2. Source data are provided as a Source Data file.

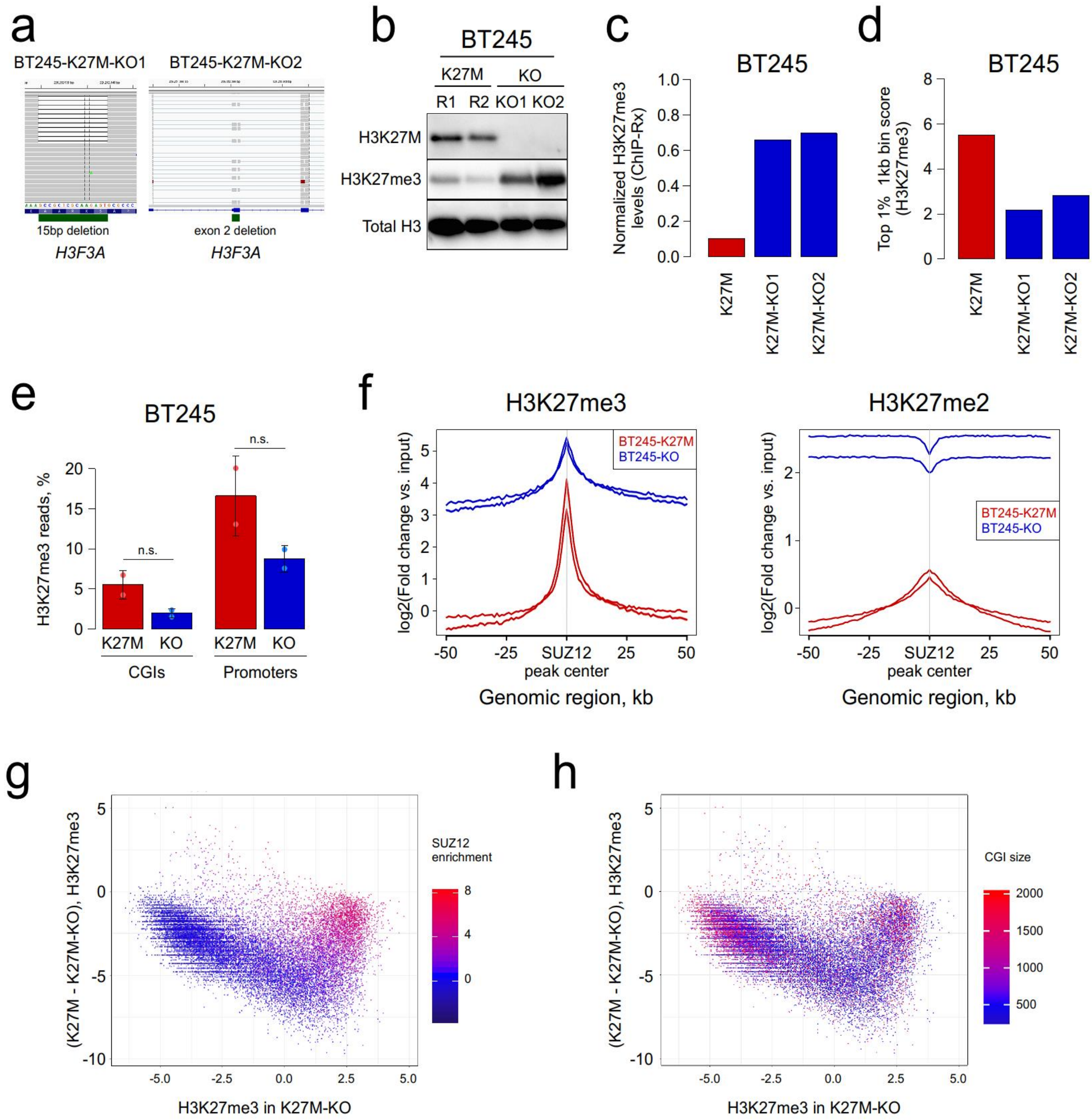

Supplementary Figure 10. CRISPR/Cas9 editing of K27M in BT245 restores increased H3K27me3 levels and spread of the mark from the unmethylated CGIs it is confined to in the presence of the mutation. **a.** MiSeq confirmation of CRISPR editing **b**. Western blot showing removal of H3K27M and increase in H3K27me3 levels **c.** ChIP-Rx scores and **d**. Top 1% 1kb bin scores showing increased H3K27me3 deposition in gene-edited lines (blue). **e.** Proportion of H3K27me3 reads in CGIs and promoters (n=2, mean ± standard deviation, Student's t-test). **f**. SUZ12 peak centered H3K27me3 and H3K27me2 aggregate plots. **g-h.** H3K27me3 levels in CGIs in BT245 (please refer to Figure 2G). For each CGI, H3K27me3 levels in K27M-KO state (x axis) are plotted against H3K27me3 change (K27M – K27M-KO) (y axis). Color coding is respectively by: (**g**) SUZ12 enrichment or (**h**) CGI size. Source data are provided as a Source Data file.

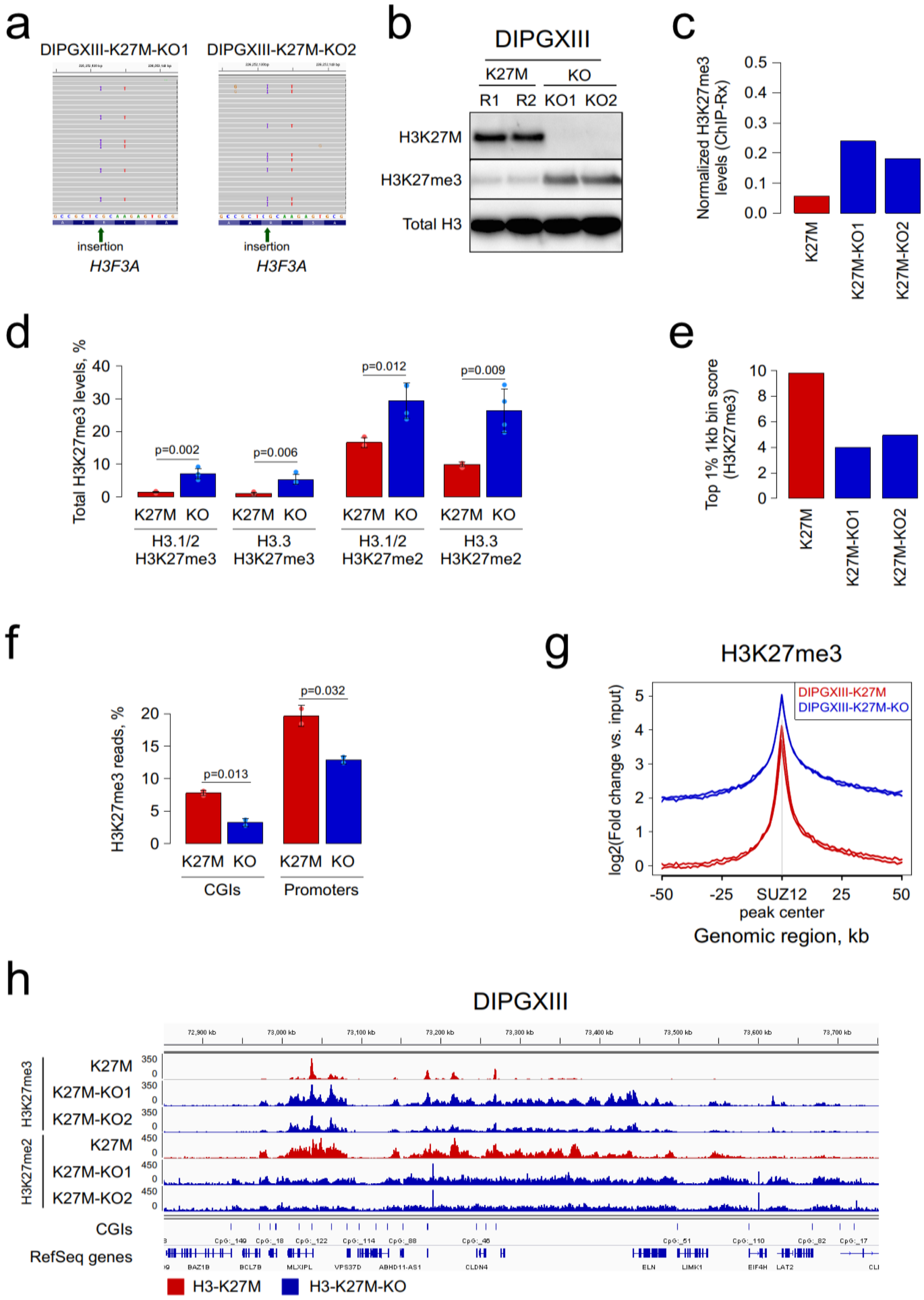

Supplementary Figure 11. CRISPR/Cas9 editing of K27M in DIPGXIII. **a.** MiSeq confirmation of CRISPR editing. **b**. Western blot showing removal of H3K27M and increase in H3K27me3 levels **c.** ChIP-Rx scores for H3K27me3. **d**. Mass spectrometry data for H3K27me2 and H3K27me3 (K27M: n=3, KO: n=4, mean ± standard deviation, Student's t-test). **e**. Top 1% 1kb bin scores for H3K27me3. **f.** Proportion of H3K27me3 reads in CGIs and promoters (n=2, mean ± standard deviation, Student's t-test). **g.** SUZ12 peak centered H3K27me3 aggregate plot for DIPGXIII **h.** ChIP-Rx normalized tracks for DIPGXIII. Source data are provided as a Source Data file.

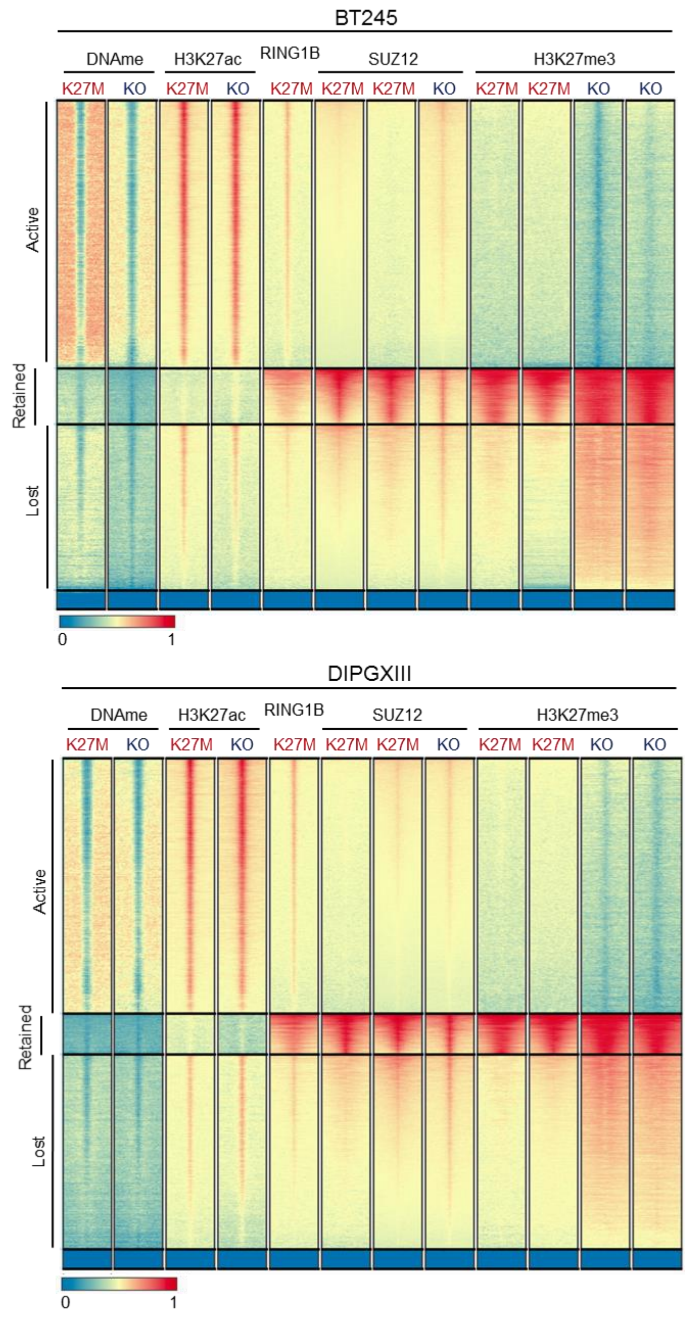

Supplementary Figure 12. Stacked heatmap plots of genome wide ChIPseq of H3K27me3, H3K27ac, SUZ12, RING1B and DNA methylation assessed using whole genome bisulfite sequencing. Note that H3K27me3 levels are unchanged in 1) sites that were previously devoid of the mark and that potentially mark active chromatin as shown by enrichment in H3K27ac in both H3K27M and H3K27-KO BT245 or DIPGXIII 2) or sites that had the mark in H3K27M cell lines (Retained). Sites that had lost the mark regain it when the mutation is removed (Lost) while limited sites show de novo gain of the mark in H3K27M cells. Two distinct edited clones are shown for each cell line.

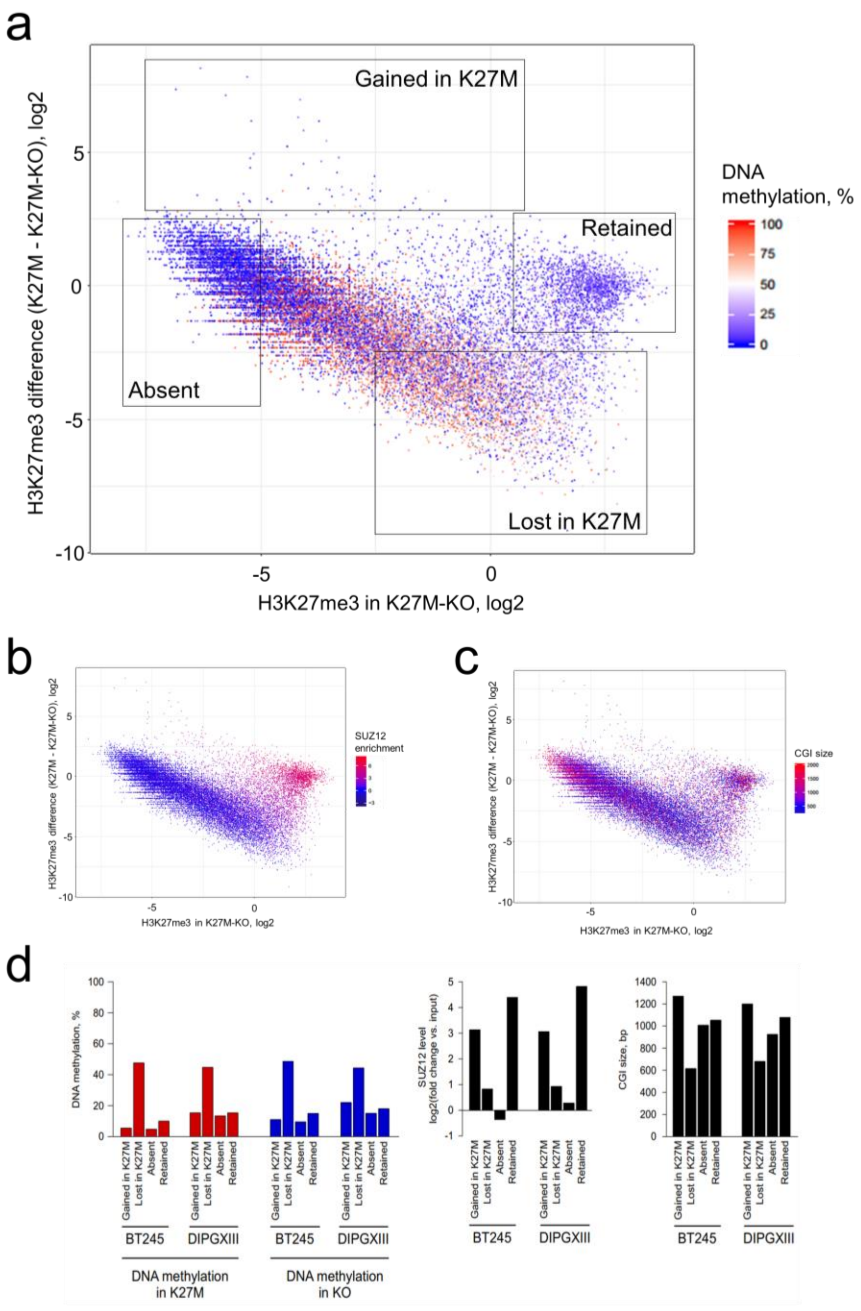

Supplementary Figure 13. H3K27me3 levels in CGIs in DIPGXIII showing similar distribution to BT245 (please refer to Fig. 2g). For each CGI, H3K27me3 levels in K27M-KO state (x axis) are plotted against H3K27me3 change (K27M – K27M-KO) (y axis). Color coding is respectively by: (**a**) DNA methylation levels as assessed using whole genome bisulphite sequencing, (**b**) SUZ12 enrichment or (**c**) CGI size. **d**. Summary statistics for Fig. 2g, Supplementary Fig. 10g-h (BT245) and 13a-c (DIPGXIII). For each category of CGIs, as depicted on Fig. 2g and Supplementary Fig. 13a, average levels of respective factors at those CGIs are shown: DNA methylation in K27M condition (left panel, in red), DNA methylation in K27M-KO condition (left panel, in blue), SUZ12 enrichment (middle panel), CGI size (right panel). In summary, DNA methylation is much higher in CGIs of “Lost in K27M” category, SUZ12 is highly enriched in “Retained” and “Gained in K27M” categories, while CGIs are smaller in “Lost in K27M” category. Source data are provided as a Source Data file.

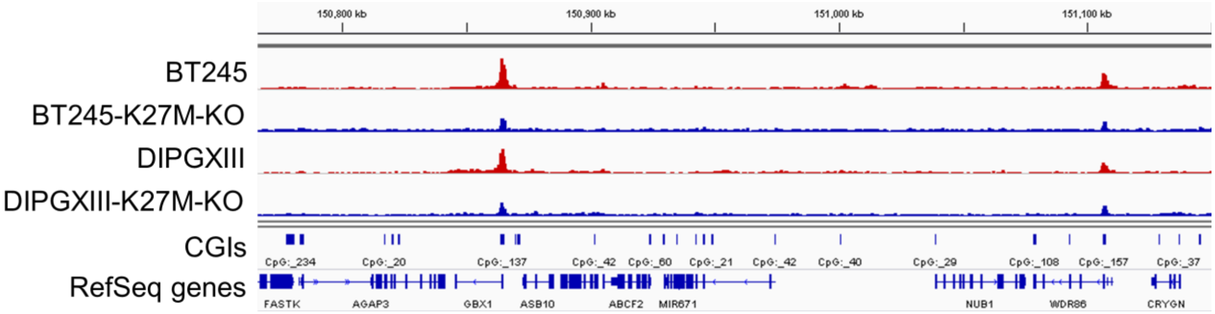

Supplementary Figure 14. SUZ12 in CRISPR-KO cells (blue tracks) vs. parental (red tracks), read depth normalized shows decreased deposition of the Polycomb Repressive Complex 2 (PRC2) complex member in gene-edited lines compared to the isogenic K27M mutant lines.

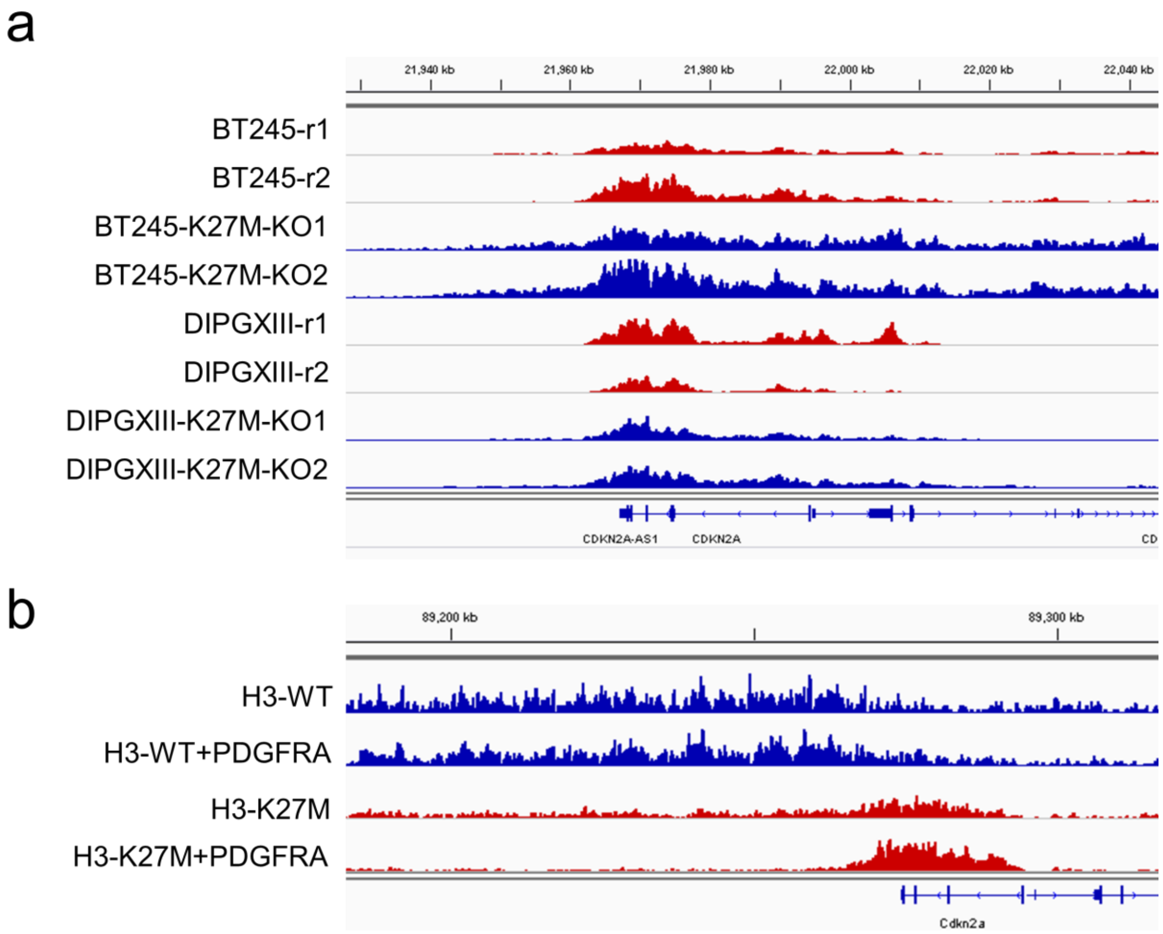

Supplementary Figure 15. **a**. Normalized H3K27me3 tracks for *CDKN2A* locus in BT245 and DIPGXIII (parental, red tracks, CRIPSR-KO, blue tracks). HEK293 and G477 tracks are not shown since both cell lines have homozygous deletion of this gene. **b**. Normalized H3K27me3 tracks for mouse H3-WT (blue) and H3-K27M (red) neural progenitor cell lines showing a degree of enrichment of H3K27me3 on the *CDKN2A* promoter. Additionally, these cells have TP53 knockout and ATRX knockdown. Two of the cell lines overexpress PDGFRA, which interestingly increases deposition of the mark on *CDKN2A* promoter in both H3K27M mutant and WT neural progenitor cells (NPC), which may account for the discrepancy with Mohammad et al. (2017)[2], where mouse NPCs with overexpression of both K27MH3.3 and *Pdgfrb* were studied. Mouse data taken from Pathania et al. (2017)[3].

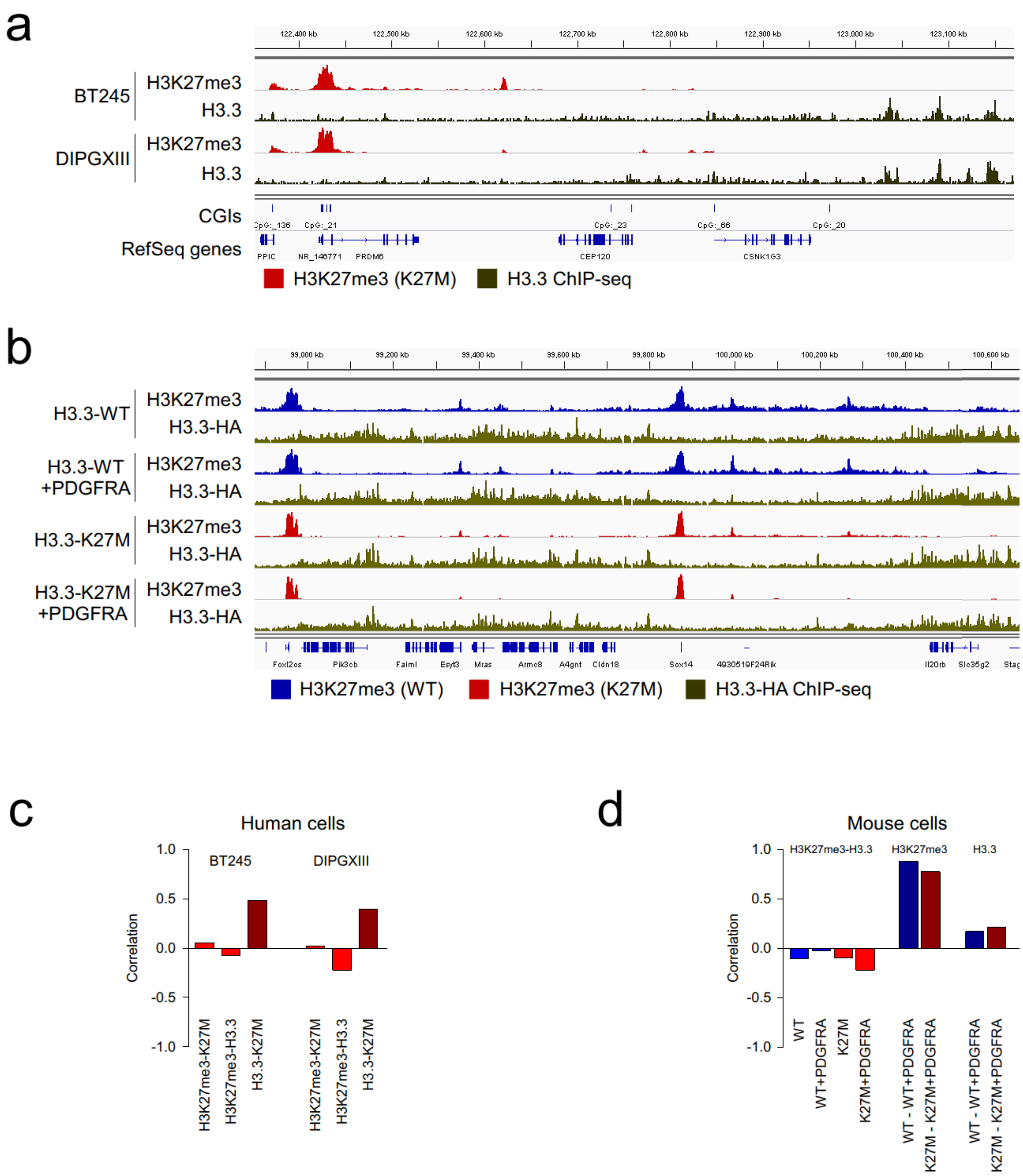

Supplementary Figure 16. H3K27me3 deposition does not correlate with K27M-H3.3 or WT-H3.3 deposition. **a.** Tracks for HGG cell lines **b.** Tracks for mouse NPC cells used in Pathania et al. (2017)[3] where the K27M-H3.3 mutant was HA-tagged. **c.** Correlation values for human cell lines. **d.** For mouse cell lines. Source data are provided as a Source Data file.

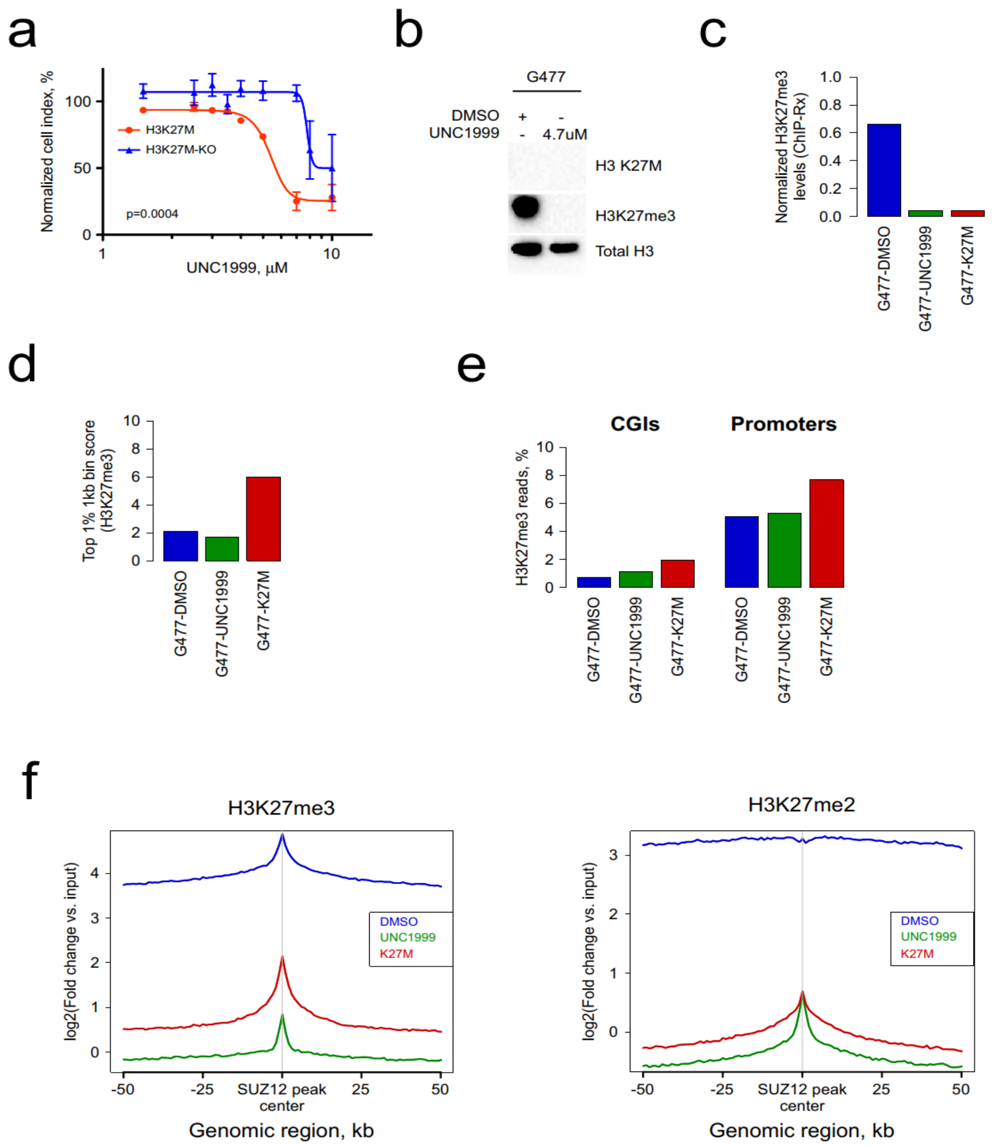

Supplementary Figure 17. High-grade glioma cell line treated with an EZH2 inhibitor. **a.** H3K27M mutant cells (BT245, red) are more sensitive to the inhibitor (UNC1999) than CRISPR-edited H3K27M-KO (blue) cells (n=3 passage replicates, mean ± standard deviation, Extra sum-of-squares F test). Similar results were observed for another inhibitor (GSK343). **b.** Drastic decrease in H3K27me3 levels in WT HGG line G477 shown by Western blot and **c.** ChIP-Rx normalized levels of H3K27me3. **d**. Top 1% 1kb bin scores for H3K27me3. **e.** Proportion of H3K27me3 reads in CGIs and promoters show global genomic decrease of H3K27me3 in G477 and no redistribution to CGIs in EZH2-inhibited cells when compared to the same line engineered to express H3.3K27M. **f.** SUZ12-centered aggregate plots of H3K27me3 and H3K27me2. Source data are provided as a Source Data file.

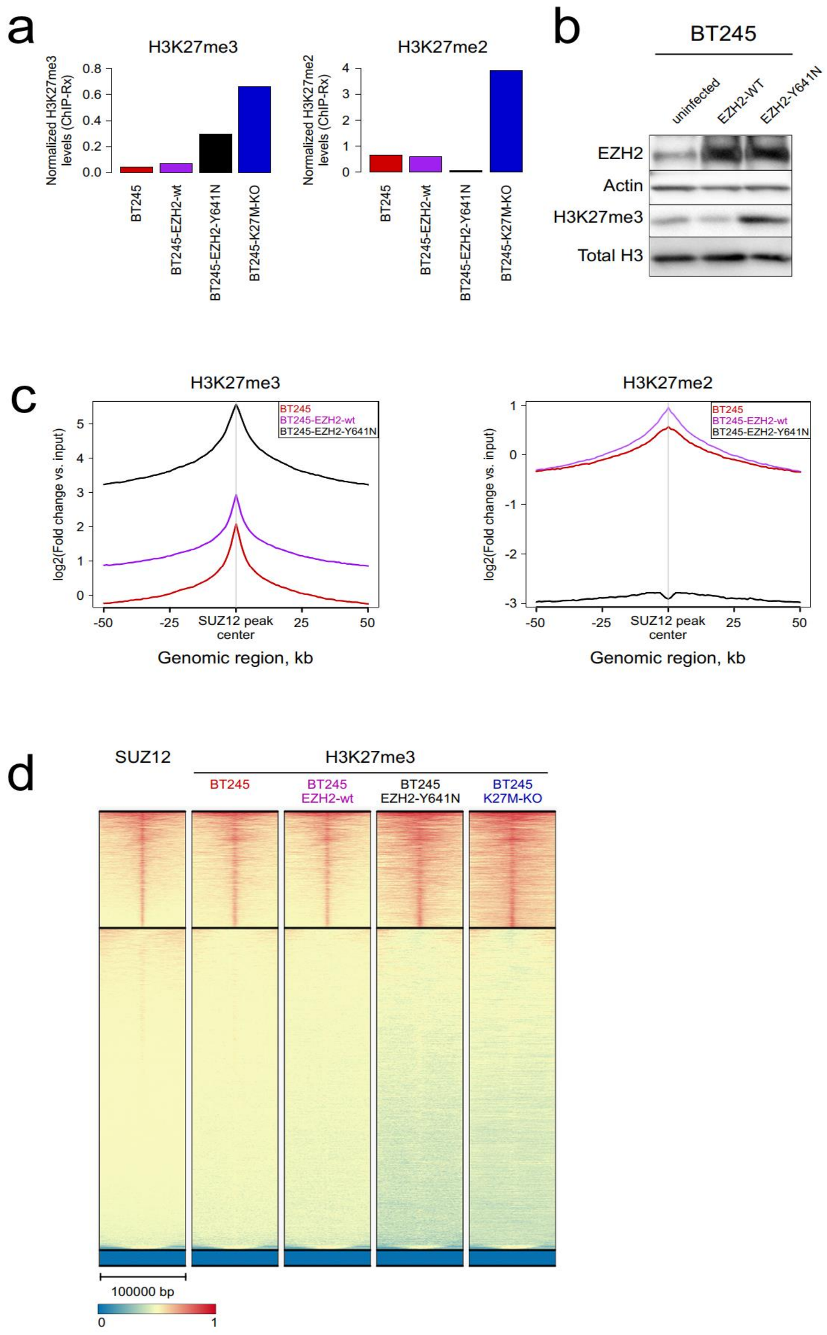

Supplementary Figure 18. H3K27me3 levels increase in K27M-mutant BT245 high-grade glioma cell line mainly when overexpressing Y641N EZH2, a mutant EZH2 shown to be less sensitive to K27M inhibition, in contrast to wild-type EZH2. This increase is accompanied in these cells by a partial restauration of the spread of the mark compared to gene-edited BT245-KO lines. Spread occurs downstream from the unmethylated CGIs it was restricted to by the H3K27M mutation. **a**. ChIP-Rx normalized H3K27me2 and H3K27me3 levels. **b.** Western blot. **c.** SUZ12 peak centered aggregate plots of H3K27me2 and H3K27me3. **d.** Heatmap plot of H3K27me3. Source data are provided as a Source Data file.

a

Gene	G477		BT245		DIPGXIII	
	LOG2FC (K27M/K27R)	P value	LOG2FC (K27M/KO)	P value	LOG2FC (K27M/KO)	P value
ID1	0.94	2.32E-17	0.97	1.18E-01	0.36	4.21E-01
ID2	0.68	**7.04E-06**	1.13	**3.34E-02**	1.75	**3.97E-07**
ID3	0.22	**4.30E-02**	1.63	**2.11E-03**	0.79	**2.41E-02**
ID4	0.78	**1.22E-06**	1.71	**2.90E-06**	1.44	**5.61E-05**

b

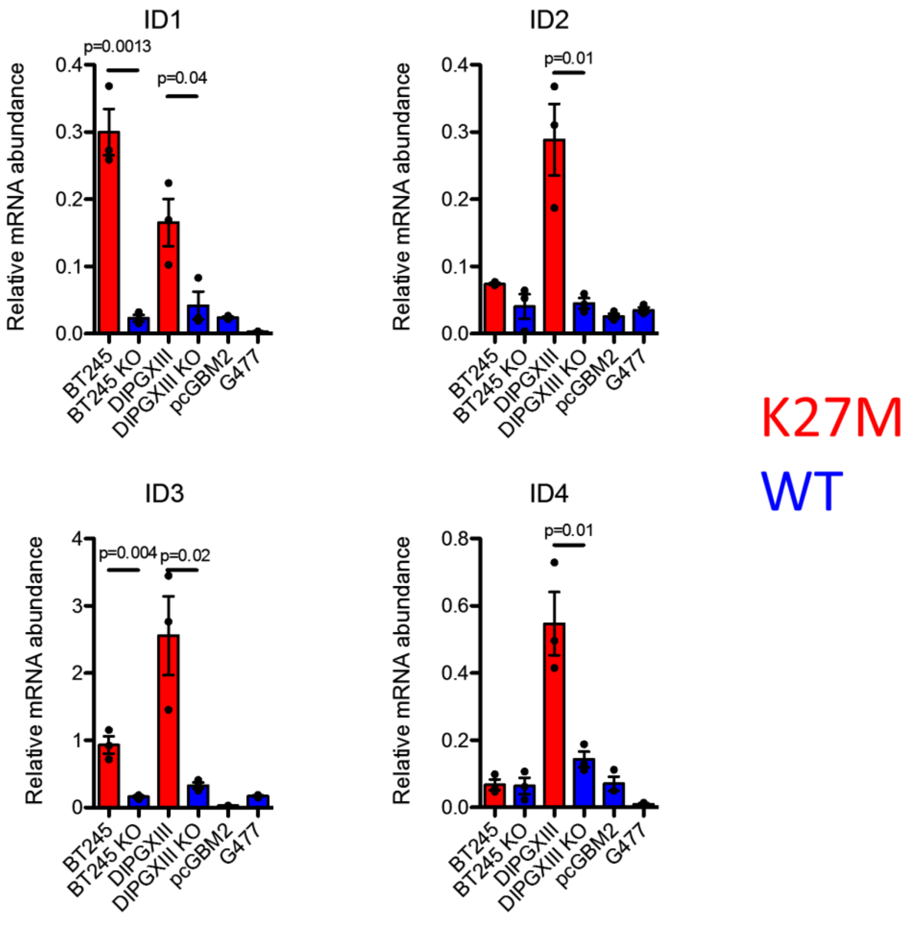

Supplementary Figure 19. ID1-4 mRNA expression in K27M HGG mutant lines (n=2 cell lines), K27M-KO controls (n=2 cell lines), and wild-type (WT) lines (n=2 cell lines), measured by **a.** RNA-seq (G477, n=3 replicates in each group; BT245, n=6 replicates in each group; DIPGXIII, n=2 replicates in each group; Wald test, p values adjusted for multiple testing), **b.** droplet digital PCR. ID genes are broadly increased in K27M compared to WT HGG lines (n = 3 passage replicates per cell line, mean ± standard error, Student's t-test comparing each K27M vs KO group). Source data are provided as a Source Data file.

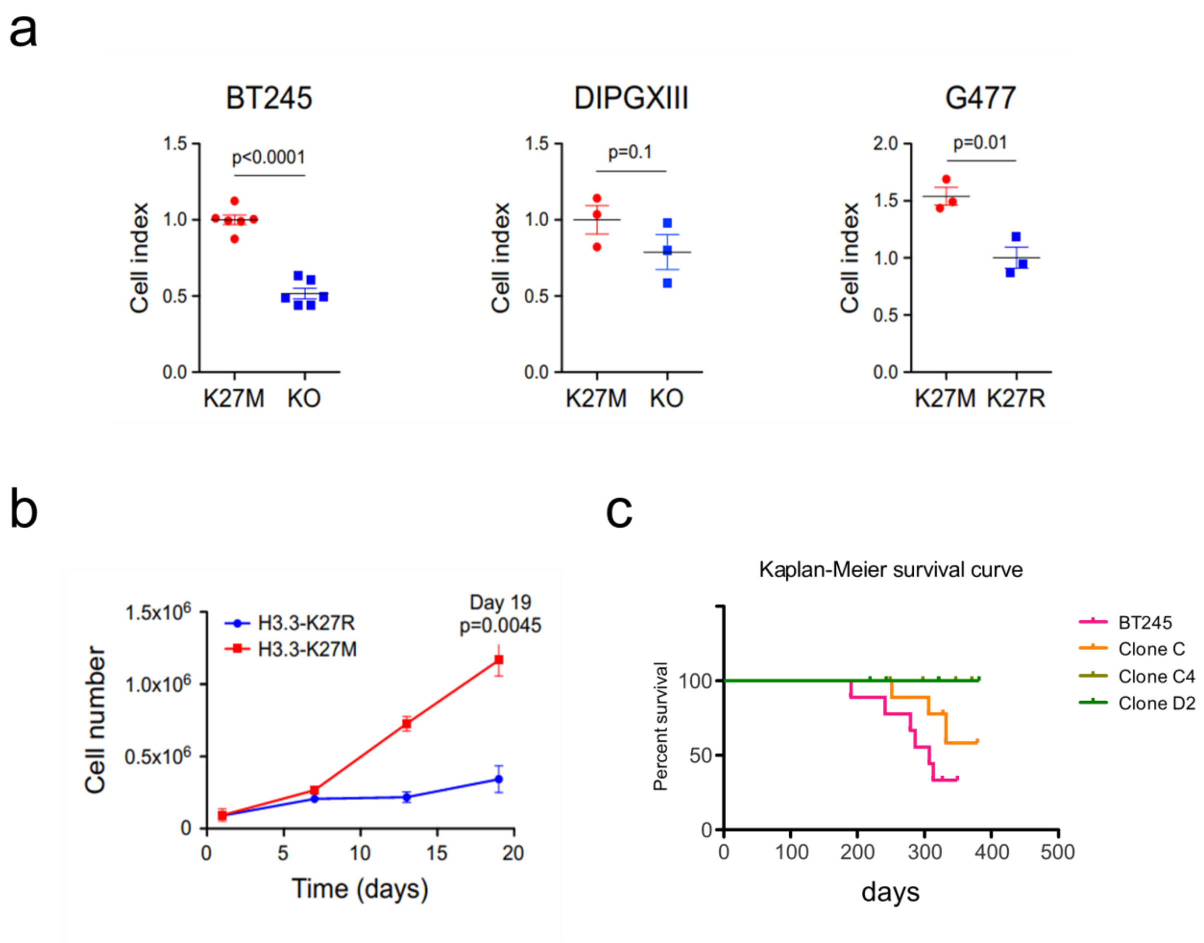

Supplementary Figure 20. **a.** Cell index in a seven-day proliferation assay of two H3K27M mutant cell lines (BT245, n=6 passage replicates in each group, DIPGXIII, n=3 passage replicates in each group) upon removal of the mutation, and a wild-type cell line (G477) upon H3.3 K27R and K27M overexpression (n=3 passage replicates in each group) (mean ± standard error, Student's t-test). **b.** Cell number over time upon rescue of BT245 H3K27M-KO lines by overexpressing H3.3-K27M or H3.3-K27R (n=3 passage replicates in each group, mean ± standard error, Student's t-test of Day 19 data). **c.** Kaplan-Meier survival curve of mice bearing orthotopic xenografts of the cell line BT245. The group bearing the parental cell line expressing K27M is shown in pink, a clone derived from CRISPR editing that maintained K27M in orange, and two clonal lines that underwent K27M knockout are shown in shades of green. (n=10 mice in Clone D2 group, n=9 mice in each of the other groups, were used for each experimental group). Source data are provided as a Source Data file.

Supplementary Table 1. List of glioblastoma cell lines and tumors used in the study.

Sample name	Cell line / tumor	Diagnosis	Histone mutation	Age	Gender	Location	Origin
BT245	Cell line	HGG	H3.3 K27M	8	M	Thalamus	K. Ligon
BT416	Cell line	HGG	H3.3 K27M	16	F	Thalamus	K. Ligon
SU-DIPGVI	Cell line	DIPG	H3.3 K27M	7	F	Pons	M. Monje
SU-DIPGXIII	Cell line	DIPG	H3.3 K27M	6	F	Pons	M. Monje
HSJ019	Cell line	HGG	H3.3 K27M	13	F	Thalamus	This study
HSJ031	Cell line	DIPG	H3.3 K27M	9	F	Pons	This study
G477	Cell line	HGG	WT	15	F	Cortex	This study
SU-pcGBM2	Cell line	HGG	WT	15	M	Cortex	M. Monje
HSJ019	Tumor	HGG	H3.3 K27M	13	F	Thalamus	This study
PS113293	Tumor	HGG	H3.3 K27M	11	M	Thalamus	This study
PH1157	Tumor	HGG	H3.3 K27M	12	M	Ventricle	This study
JN57	Tumor	DIPG	H3.3 K27M	n/a	M	Pons	This study
BTTB434	Tumor	HGG	WT	9	F	Cortex	This study
1840	Tumor	HGG	WT	73	F	Cortex	This study
2230	Tumor	HGG	WT	72	M	Cortex	This study

Supplementary Table 2. ChIP-Rx normalization values, reflecting total H3K27me3 levels.

**Sample**	Histone mark	ChIP-Rx value
BT245-K27M1	H3K27me3	0.043
BT245-K27M2	H3K27me3	0.102
BT245-K27M-KO1	H3K27me3	0.661
BT245-K27M-KO2	H3K27me3	0.697
BT245-EZH2-wt	H3K27me3	0.070
BT245-EZH2-Y641N	H3K27me3	0.295
BT245-UNC1999	H3K27me3	0.052
DIPGXIII-K27M	H3K27me3	0.056

DIPGXIII-K27M-KO1	H3K27me3	0.239
DIPGXIII-K27M-KO2	H3K27me3	0.181
DIPGXIII-DMSO	H3K27me3	0.048
DIPGXIII-UNC1999	H3K27me3	0.019
G477-DMSO	H3K27me3	0.663
G477-UNC1999	H3K27me3	0.041
G477-K27R	H3K27me3	0.960
G477-K27M	H3K27me3	0.040
pcGBM2	H3K27me3	0.438
HSJ019	H3K27me3	0.113
HSJ019-Tumor	H3K27me3	0.244
HEK293T	H3K27me3	0.629
HEK293T-H3.1K27M	H3K27me3	0.044
H1	H3K27me3	0.034
BT245-K27M1	H3K27me2	0.656
BT245-K27M2	H3K27me2	0.720
BT245-K27M-KO1	H3K27me2	4.433
BT245-K27M-KO2	H3K27me2	3.905
BT245-EZH2-wt	H3K27me2	0.593
BT245-EZH2-Y641N	H3K27me2	0.062
BT245-UNC1999	H3K27me2	0.294
DIPGXIII-K27M	H3K27me2	0.640
DIPGXIII-K27M-KO1	H3K27me2	1.115
DIPGXIII-K27M-KO2	H3K27me2	1.156
G477-DMSO	H3K27me2	2.284
G477-UNC1999	H3K27me2	0.205
G477-K27R	H3K27me2	1.462
G477-K27M	H3K27me2	0.225
pcGBM2	H3K27me2	1.284
HSJ019	H3K27me2	0.922

Supplementary Table 3. List of genes with H3K27me3 gain at promoters in K27M condition compared to non-K27M.

**BT245 (n=245)**	**G477 (n=163)**	**DIPGXIII (n=64)**	**Overlap**
POU3F2	ATF3	RUNX1T1	BC014312 (BT245-G477)
DLGAP1	CDK6	ADAMTS9	C1orf61 (BT245-G477)
MAP4K5	SULF2	DSC3	HIST1H2AG (BT245-G477)
FAM46A	SOX21	MBD1	HIST1H2BJ (BT245-G477)
SFMBT1	HIC1	PLEKHG4	MIR9-1 (BT245-G477)
IGF2R	IGF2BP3	GNL3	SULF2 (BT245-G477)
SOBP	PIK3R3	CXXC1	ZNF75A (BT245-G477)
TDRD7	SYDE2	DNAJB5	
ATL1	SMURF2	NRN1	UNC5C (BT245-DIPGXIII)
SUSD5	ECH1	MANF	
VWCE	PTOV1	SRRD	
NKX2-2	NAB1	ZFHX4	
KCND2	CD2BP2	SMPD4	
MYO5A	ZEB2	MZT2B	
FBN1	ST6GALNAC6	RASEF	
PROM1	BOLA2	EPHA5	
PLEKHH1	ZNF439	ZNF532	
EPB41L4A	TNK2	BCR	
SH3RF3	L2HGDH	EYA4	
UGP2	HIST1H1E	NEDD4L	
RBPMS	CALM2	PRPF40A	
TMEM163	NR6A1	CFL1	
SGK1	ST20-MTHFS	MBIP	
LBX2-AS1	RNF220	PCOLCE	
FAM5B	PTBP2	MEOX2	
POPDC3	CBX8	UNC5C	
PDGFRA	C1orf61	MALAT1	
SOWAHA	ZNF528	OTX1	
PDE6A	FOXG1	HPS4	
SLC1A1	H2AFJ	TRHDE	
BVES-AS1	MAPK9	IQUB	
TMEM229B	HOXB6	PBRM1	
SLC26A4	ZNF623	ETV1	
C8orf44-SGK3	TNFRSF19	ARL6IP6	
SGK3	HIST1H2BJ	ZBTB20	
ERBB3	ZNF462	PISD	
SLC24A3	ZNF428	EIF4ENIF1	
SIX1	TBC1D10B	TRHDE-AS1	

GAS2	HOXB7	FAM5C	
BVES	ZNF100	PAK2	
NRIP3	ZNF605	FLJ14346	
ITGA9	CDK10	EBF1	
OSR2	DDX19A	CRYBB2P1	
IGFBP1	RICTOR	RBMS1	
SIX2	ZNF274	LRP5L	
IGSF9B	ZNF766	FBXW4P1	
UGT8	PELO	RBM15B	
ACVR1	LOC100131691	AQPEP	
YPEL2	MECOM	MUS81	
PPP2R2B	POU3F3	HEY2	
EXTL1	FGF11	JPH1	
SFRP1	CCND2	ZADH2	
BMP8B	CTBP1	AX747550	
CLIP4	FN3K	LOC100132215	
ITGB3	ITGA1	STC2	
ZNF567	PLAG1	ZFHX4-AS1	
MEG3	GATA2	PCOLCE-AS1	
ITGB8	DDX19B	CR936711	
PCDHGA9	SOLH	ADAMTS9-AS2	
LARGE	ZZEF1	AK123543	
CTTNBP2	METTL8	LOC100144602	
LBX2	ZNF587	BC093903	
LDLRAD4	HOXB9	MIR4785	
SNTB1	ZNF75A	BC036196	
VLDLR	AFG3L1P		
43351	ZNF8		
CLTC	ZSCAN12		
AKNAD1	ZNFX1		
HRASLS	ZNF583		
FLJ11235	RBM20		
PCDHGA2	ZNF506		
HAS2	ZNF271		
LHFPL3	MIB2		
ANKRD44	TMEM102		
PCDHGC3	EPHA7		
ATP11A	TSSC4		
MDGA2	BBC3		
GPSM2	ZNF587B		
PCDHGB3	MEIS1		

PCDHGB6	HSPD1		
PCDHGA1	AB209061		
PCDHGA11	ZNF596		
APBB2	CLASP1		
PCDHGB4	ZSCAN22		
PCDHGC4	CTBP1-AS1		
PCDHGB5	ZSCAN30		
C3orf70	CYB5D2		
PCDHGB7	HNRNPL		
PCDHGA7	ZNF620		
AK4	MZF1		
PCDHGA10	CHCHD7		
PCDHGA8	HOXB8		
PCDHGA3	MGC2752		
PCDHGA4	HIST1H2BD		
PCDHGA6	DGKK		
PCDHGA12	ZNF582		
PCDHGA5	ZNF562		
GRIK3	ZNF286B		
PCDHGB1	C1orf174		
PCDHGB2	ST20		
SULF2	ZNF701		
LEF1	HIST1H2AG		
TTC39C	ZFP28		
SMOC1	PRRT2		
PCDHGC5	C2orf61		
FAM149A	DCAF17		
TMEM154	ZNF576		
FTCDNL1	MINK1		
VAX2	RPL12		
MYC	IGF2BP1		
MYT1	MMP23B		
ACOXL	RPL23AP53		
PHACTR2	CENPBD1		
TACC2	UBE2S		
CACNA1E	ZNF383		
LGR5	PPP2R1A		
EFHD1	MAZ		
FZD6	SRRM5		
ZNF75A	LHX1		
ZBTB16	C17orf107		

CD302	CHRNE		
PKNOX2	PRDM12		
ATF7IP	HOXB-AS3		
LY75-CD302	HOXB5		
PCDH1	IRS4		
ZFYVE28	LIN28B		
OLFML1	SNORD12B		
SH3RF3-AS1	SNORD12		
BAMBI	SNORD12C		
RAMP1	ZFAS1		
EGR3	FOXO3B		
CCDC122	MIR643		
CELF2	LOC641367		
HECW1	BC031657		
NFATC1	BC014312		
ANGPT1	LOC254128		
AK125994	LOC100506421		
HIST1H2BJ	ZNF582-AS1		
CDC42EP1	BC063675		
PCGF1	MEIS1-AS3		
ARHGAP20	ZNF790-AS1		
ATRNL1	AK128697		
TRIM67	PTOV1-AS1		
KIAA1244	BC084573		
FLJ10038	EVI1		
GRIK4	MIR9-1		
BMP2	LOC100133612		
PCDH10	ZEB2_AS1_3		
C1orf61	ZEB2-AS1		
FAM198A	ZEB2_AS1_4		
STOX2	AY343891		
SNX10	AK055459		
HIST1H2AG	MIR3190		
FAM155B	MIR3191		
GABPB1	BC047644		
ICA1	AGRP		
GRHL1	C4orf42		
TEX15	LINC00235		
SALL3	ZEB2_AS1_1		
GPR137B	AK097590		
PLD5	BX537909		

DGKD	SNORA65		
SLC22A23	AK097472		
ZNF141			
TRIM71			
PPFIBP2			
IGSF11			
WIBG			
KCNA3			
HIST1H4F			
SHISA7			
UBE3A			
NTRK3			
STK3			
LACC1			
PCDH9			
SNX29P1			
UNC5C			
TCF7L1			
ZNF470			
RUNX1			
BCAN			
TSPAN8			
TBR1			
LOC402160			
SP9			
NALCN			
SAG			
GNGT1			
TFPI2			
LOC100128750			
IGFBP3			
MIR9-1			
HAS2-AS1			
MIR4697HG			
JB175200			
BC051708			
LEF1-AS1			
U4			
MIR1206			
PVT1			
PGM5P2			

BC035370			
LOC283683			
NTRK3-AS1			
SNORD116-4			
IPW			
BC005081			
TPTEP1			
SNORD116-10			
MGC2889			
SNORD116-2			
SNORD116-3			
BC039551			
SNORD116-5			
BC041855			
SNORD116-8			
MEG3_1			
SLC26A4-AS1			
MIR4764			
MIR377			
MIR541			
MIR409			
MIR410			
MIR412			
MIR656			
MIR369			
FLJ35024			
LINC00643			
SNORD116-11			
BC014312			
BC040219			
BX647249			
AK096549			
MIR1204			
AIRN			
ANKRD18DP			
MIR154			
MIR496			
SNORD116-12			
LINC00623			
AB074166			
LINC00869			

GABPB1-AS1			
BX537481			

Supplementary Table 4. List of differentially expressed genes, overlapping across different datasets.

**Up in K27M (BT245, DIPGXIII) (n=102)**	**Up in K27M (BT245, G477) (n=9)**
ABCB1	ADAMTS1
ACSBG1	AKR1C3
ADAMTS12	COL6A3
ADAMTS19	LPL
ADARB2	MGST1
ADCY2	PKIB
ADCYAP1R1	RYR2
AFF3	STXBP6
ANK2	UNC5B
ASS1	
ATP2A3	**Up in K27M (DIPGXIII, G477) (n=17)**
B3GALTL	ALDH1L1
BCL11B	ANTXR2
BTBD17	ATP9A
C10orf107	CPS1
C14orf132	FRAS1
C8orf4	FRY
CACHD1	FXYD6
CCDC102B	FXYD6-FXYD2
CD70	HMCN1
CDHR3	MEGF6
CHL1	PARD3B
CNTN4	PCSK2
CRB2	PLCB4
CXCL2	SLC44A5
DAAM2	SNX10
DOK5	THSD7A
EFNA5	UNC5D
EML5	
F3	**Down in K27M (BT245, DIPGXIII) (n=12)**
FAM198B	BCAS1
FILIP1	ELFN2
FLRT3	GAL3ST1

HECW2	GPX3
HEY1	ICAM1
HLA-DPA1	JSRP1
HSD11B2	NRIP3
HTRA1	SERPINF1
ID2	SHROOM1
ID4	SNPH
IGFBPL1	SQRDL
IGSF9	ZBTB8B
IL33	
INPP5D	**Down in K27M (BT245, G477) (n=2)**
IRX1	DAPK2
ITGA6	SPNS2
JAM2	
KAL1	**Down in K27M (DIPGXIII, G477) (n=0)**
KATNAL2	
KAZN	
KCNK10	
KIRREL2	
LAMA2	
LAMP5	
LFNG	
LRRC3B	
MAPK15	
MFNG	
MGARP	
NAALAD2	
NDP	
NES	
NKAIN3	
NOS2	
NREP	
PALMD	
PAM	
PAPLN	
PCDH18	
PDE3A	
PDE5A	
PDE8B	
PELI2	
PI15	

PKP2	
PLK2	
POU3F4	
PREX2	
PTPRM	
RASA4	
RASA4B	
RFX4	
ROBO2	
SGK223	
SH3GL2	
SLC16A9	
SLC19A3	
SLC1A3	
SLC4A4	
SLC8A1	
SLIT2	
ST8SIA4	
STXBP5L	
TMEM200C	
TMEM47	
TMEM74	
TNNI3K	
TPPP3	
TRIM22	
TRPC4	
UG0898H09	
ZNF311	

Supplementary Table 5. Sequences of single guide RNA targets and repair template.

H3F3A_K27M	GAGGGCGCACTCATGCGAG
HIST1H3B_WT	GGCTGCTCGCAAGAGCGCGC
HIST1H3B_WT_to_K27M	ACTTTTGGTAGCGGCGGATCTCGCGCAGAGCCACAGTGCC CGGGCGGTAACGGTGAGGCTTTTTCACGCCGCCGGTAGCT GGCGCGCTCATGCGAGCAGCCTTGGTAGCCAGCTGCTTG CGTGGCGCTTTACCGCCGGTGGATTTCCGAGCTGTCTGTT TAGTACGAGC

Supplementary Table 6. List of antibodies used in this study.

	Experiment	Company, cat. no	Dilution
Total H3	WB	Abcam 1791	1:2000
H3K27me3	WB	Millipore ABE44	1:1000
H3K27M	WB, ChIP-seq	Millipore ABE419	1:200 (WB)<br>1:66 (ChIP-seq)
EZH2	WB	Cell Signaling #5246	1:1000
Beta-actin	WB	Cell Signaling #4970	1:1000
H3K27me3	ChIP-seq	Cell Signaling #9733	1:40
H3K27me3	ChIP-seq	Active Motif #61017	1:100
H3K27me2	ChIP-seq	Cell Signaling #9728	1:50
H3.3	ChIP-seq	Millipore 09-838	1:66
SUZ12	ChIP-seq	Cell Signaling #3737	1:150
RING1B	ChIP-seq	Active Motif 39663	1:200
HA	ChIP-seq	Cell Signaling # 3724	1:100

Supplementary Table 7. Primer sequences of digital PCR targets

Gene	exon location	Forward	Reverse
GAPDH	2-3	tgtagttgaggtcaatgaaggg	acatcgctcagacaccatg
ID1	1-1	ctccttgccccctggatg	tcaacggcgagatcagc
ID2	1-3	cttaaagattccgtgaatttgttgt	atcagcatcctgtccttgc
ID3	3-3	cgcattgttacagaaagtcacc	tgctctccaaactatgccaag
ID4	3-3	acagtagcttagcgtaacatagc	cataatggcaaatccttcaagca

Supplementary References

1 Lewis, P. W. *et al.* Inhibition of PRC2 activity by a gain-of-function H3 mutation found in pediatric glioblastoma. *Science* **340**, 857-861, doi:10.1126/science.1232245 (2013).

2 Mohammad, F. *et al.* EZH2 is a potential therapeutic target for H3K27M-mutant pediatric gliomas. *Nature medicine* **23**, 483-492, doi:10.1038/nm.4293 (2017).

3 Pathania, M. *et al.* H3.3(K27M) Cooperates with Trp53 Loss and PDGFRA Gain in Mouse Embryonic Neural Progenitor Cells to Induce Invasive High-Grade Gliomas. *Cancer cell* **32**, 684-700 e689, doi:10.1016/j.ccell.2017.09.014 (2017).
