# Supplementary material for: H3K27M induces defective chromatin spread of PRC2-mediated repressive H3K27me2/me3 and is essential for glioma tumorigenesis
Source: Nat Commun. 2019 Mar 19;10:1262. doi: 10.1038/s41467-019-09140-x (PMC6425035; doi:10.1038/s41467-019-09140-x)
Supplement: Supplementary file 3 — Reporting Summary [file 41467_2019_9140_MOESM3_ESM.pdf]

nature research

Corresponding author(s): Nada Jabado, Jacek Majewski

Last updated by author(s): Jan 18, 2019

Reporting Summary

Nature Research wishes to improve the reproducibility of the work that we publish. This form provides structure for consistency and transparency in reporting. For further information on Nature Research policies, see Authors & Referees and the Editorial Policy Checklist.

Statistics

For all statistical analyses, confirm that the following items are present in the figure legend, table legend, main text, or Methods section.

n/a	Confirmed	
☐	☒	The exact sample size (*n*) for each experimental group/condition, given as a discrete number and unit of measurement
☐	☒	A statement on whether measurements were taken from distinct samples or whether the same sample was measured repeatedly
☐	☒	The statistical test(s) used AND whether they are one- or two-sided<br>*Only common tests should be described solely by name; describe more complex techniques in the Methods section.*
☒	☐	A description of all covariates tested
☐	☒	A description of any assumptions or corrections, such as tests of normality and adjustment for multiple comparisons
☐	☒	A full description of the statistical parameters including central tendency (e.g. means) or other basic estimates (e.g. regression coefficient) AND variation (e.g. standard deviation) or associated estimates of uncertainty (e.g. confidence intervals)
☐	☒	For null hypothesis testing, the test statistic (e.g. *F*, *t*, *r*) with confidence intervals, effect sizes, degrees of freedom and *P* value noted<br>*Give P values as exact values whenever suitable.*
☒	☐	For Bayesian analysis, information on the choice of priors and Markov chain Monte Carlo settings
☒	☐	For hierarchical and complex designs, identification of the appropriate level for tests and full reporting of outcomes
☒	☐	Estimates of effect sizes (e.g. Cohen's *d*, Pearson's *r*), indicating how they were calculated

Our web collection on statistics for biologists contains articles on many of the points above.

Software and code

Policy information about availability of computer code

Data collection	No software was used to collect data for this study.
Data analysis	ChIP-seq raw reads were aligned to human (hg19) or mouse (mm10) and drosophila (dm6) genome build using BWA version 0.7.17 with default parameters. Read counting in bins was done using bedtools version 2.22.1. ChIP-sequencing coverage tracks were visualized using IGV 2.3 software. Peak-calling was done using MACS2 version 2.1.1. The SUZ12 peak-centered H3K27me3 enrichment plots and PMD plot were generated using ngs.plot.r package. The aggregate plots of SUZ12 and H3K27me3 were generated using deepTools v3.1.0. Heat map plots for comparing H3K27me3, SUZ12, RING1B, and DNA methylation were generated using ChAsE v.1.0.11 software. Custom R scripts (available upon request) were used to generate the top 1% bin scores, the plots of H3K27me3 variations, the correlation plots of K27M-K27me2 and WT-K27me3 and the Decile plots.<br><br>RNA-seq raw reads were aligned to human genome build (UCSC hg19) using STAR version 2.5.3a. The mapped reads for genes were counted using featureCounts program (version 1.5.3). Gene set enrichment analysis was performed using the GSEA tool from Broad Institute.<br><br>WGBS raw reads were aligned to human genome build (UCSC hg19) using BWA (version 0.6.1). Samtools (version 0.1.18) in mpileup mode was applied to call methylation of individual CpGs.

For manuscripts utilizing custom algorithms or software that are central to the research but not yet described in published literature, software must be made available to editors/reviewers. We strongly encourage code deposition in a community repository (e.g. GitHub). See the Nature Research guidelines for submitting code & software for further information.

Data

Policy information about availability of data

All manuscripts must include a data availability statement. This statement should provide the following information, where applicable:

- Accession codes, unique identifiers, or web links for publicly available datasets
- A list of figures that have associated raw data
- A description of any restrictions on data availability

The ChIP-seq, RNA-seq and WGBS data have been uploaded to our data hub (https://datahub-jv6f4mbl.udes.genap.ca/).

Field-specific reporting

Please select the one below that is the best fit for your research. If you are not sure, read the appropriate sections before making your selection.

☒ Life sciences ☐ Behavioural & social sciences ☐ Ecological, evolutionary & environmental sciences

For a reference copy of the document with all sections, see nature.com/documents/nr-reporting-summary-flat.pdf

Life sciences study design

All studies must disclose on these points even when the disclosure is negative.

Sample size	No statistical measures were used to determine sample size.
Data exclusions	No data was excluded. All data is presented.
Replication	All attempts at replication were successful.
Randomization	Samples were allocated to groups according to genotype. No randomization was required because the sequencing of nucleic acid libraries are not affected by sample randomization.
Blinding	There were no studies in which investigators were blinded, however results were validated in biological replicates.

Reporting for specific materials, systems and methods

We require information from authors about some types of materials, experimental systems and methods used in many studies. Here, indicate whether each material, system or method listed is relevant to your study. If you are not sure if a list item applies to your research, read the appropriate section before selecting a response.

Materials & experimental systems

n/a	Involved in the study	
☐	☒	Antibodies
☐	☒	Eukaryotic cell lines
☒	☐	Palaeontology
☐	☒	Animals and other organisms
☒	☐	Human research participants
☒	☐	Clinical data

Methods

n/a	Involved in the study	
☐	☒	ChIP-seq
☒	☐	Flow cytometry
☒	☐	MRI-based neuroimaging

Antibodies

Antibodies used	For Immunoblotting, the following primary antibodies were used: anti-Total H3 (Abcam, ab1791), anti-H3K27me3 (Millipore, ABE44), anti-EZH2 (Cell Signaling Tech, 5246), anti-β-actin (Cell Signaling Tech, 4970), anti-H3K27M (Millipore, ABE419).<br><br>For ChIP, the following primary antibodies were used: anti-H3K27me3 (Cell Signaling Tech, 9733), anti-H3K27me3 (Active Motif, 61017), anti-H3K27me2 (Cell Signaling Tech, 9728), anti-SUZ12 (Cell Signaling Tech, 3737), anti-RING1B (Active Motif, 39663), anti-H3.3 (Millipore, 09-838), anti-H3K27M (Millipore, ABE419), anti-HA (Cell Signaling Tech, 3724).
Validation	For ChIP, the specificity of antibodies was tested by dot blot on a histone modification peptide array. Antibodies used have been used in the literature and also validated by manufacturers.

Eukaryotic cell lines

Policy information about cell lines

Cell line source(s)	293T (ATCC)<br>H1 (WiCell)<br>BT245 (human tumor)<br>DIPG-XIII (human tumor)<br>DIPG-VI (human tumor)<br>HSJ-019 (human tumor)<br>BT416 (human tumor)<br>G477 (human tumor)<br>pcGBM2 (human tumor)
Authentication	Cell lines have been authenticated as unique by distinct DNA methylation, RNA-seq, and chromatin patterns. The identity of cell lines were checked by microsatellite typing (DNA fingerprinting). CRISPR/Cas9 edited clones were verified by Sanger sequencing and targeted resequencing by MiSeq to confirm specific genome editing event.
Mycoplasma contamination	All cell lines tested negative for mycoplasma contamination.
Commonly misidentified lines<br>(See ICLAC register)	No commonly misidentified lines were used in this study.

Animals and other organisms

Policy information about studies involving animals; ARRIVE guidelines recommended for reporting animal research

Laboratory animals	Mus musculus, NSG mice (strain NOD.Cg-Prkdc-scid Il2rg-tm1Wjl/Sz), female, 6-8 weeks of age.
Wild animals	The study did not involve wild animals.
Field-collected samples	The study did not involve samples collected from the field.
Ethics oversight	*Identify the organization(s) that approved or provided guidance on the study protocol, OR state that no ethical approval or guidance was required and explain why not.*

Note that full information on the approval of the study protocol must also be provided in the manuscript.

ChIP-seq

Data deposition

☒ Confirm that both raw and final processed data have been deposited in a public database such as GEO.

☒ Confirm that you have deposited or provided access to graph files (e.g. BED files) for the called peaks.

Data access links<br>*May remain private before publication.*	https://datahub-jv6f4mbl.udes.genap.ca/
Files in database submission	BT245-EZH2-Y641N_H3K27me2.normalized.bw<br>BT245-EZH2-Y641N_H3K27me3.normalized.bw<br>BT245-EZH2-Y641N_Input.bw<br>BT245-EZH2-wt_H3K27me2.normalized.bw<br>BT245-EZH2-wt_H3K27me3.normalized.bw<br>BT245-EZH2-wt_Input.bw<br>BT245-K27M1_H3K27me2.normalized.bw<br>BT245-K27M1_H3K27me3.normalized.bw<br>BT245-K27M1_Input.bw<br>BT245-K27M2_H3K27me2.normalized.bw<br>BT245-K27M2_H3K27me3.normalized.bw<br>BT245-K27M2_Input.bw<br>BT245-K27M2_SUZ12.bw<br>BT245-K27M3_H3.3.bw<br>BT245-K27M3_input.bw<br>BT245-K27M4_Input.bw<br>BT245-K27M4_K27M.bw<br>BT245-K27M5_Input.bw<br>BT245-K27M5_RING1B.bw<br>BT245-K27M5_SUZ12.bw<br>BT245-KO1-r2_H3K27me2.normalized.bw<br>BT245-KO1-r2_Input.bw<br>BT245-KO1_H3K27me3.normalized.bw

BT245-KO1_Input.bw
BT245-KO2-r2_H3K27me2.normalized.bw
BT245-KO2-r2_Input.bw
BT245-KO2-r2_SUZ12.bw
BT245-KO2_H3K27me3.normalized.bw
BT245-KO2_Input.bw
BT245-UNC1999_H3K27me2.normalized.bw
BT245-UNC1999_H3K27me3.normalized.bw
BT245-UNC1999_Input.bw
BT416-K27M_H3K27me3.bw
BT416-K27M_Input.bw
DIPGVI-K27M1_H3K27me3.bw
DIPGVI-K27M1_Input.bw
DIPGVI-K27M2_Input.bw
DIPGVI-K27M2_RING1B.bw
DIPGVI-K27M2_SUZ12.bw
DIPGXIII-K27M-DMSO_H3K27me3.normalized.bw
DIPGXIII-K27M-DMSO_Input.bw
DIPGXIII-K27M-DMSO_SUZ12.bw
DIPGXIII-K27M1_H3K27me2.normalized.bw
DIPGXIII-K27M1_H3K27me3.normalized.bw
DIPGXIII-K27M1_Input.bw
DIPGXIII-K27M1_K27M.bw
DIPGXIII-K27M2_H3.3.bw
DIPGXIII-K27M2_Input.bw
DIPGXIII-K27M3_Input.bw
DIPGXIII-K27M3_RING1B.bw
DIPGXIII-K27M3_SUZ12.bw
DIPGXIII-KO1_H3K27me2.normalized.bw
DIPGXIII-KO1_H3K27me3.normalized.bw
DIPGXIII-KO1_Input.bw
DIPGXIII-KO1_SUZ12.bw
DIPGXIII-KO2_H3K27me2.normalized.bw
DIPGXIII-KO2_H3K27me3.normalized.bw
DIPGXIII-KO2_Input.bw
DIPGXIII-UNC1999_H3K27me3.normalized.bw
DIPGXIII-UNC1999_Input.bw
G477-DMSO2_H3K27me2.normalized.bw
G477-DMSO2_Input.bw
G477-DMSO3_H3K27me2.normalized.bw
G477-DMSO3_Input.bw
G477-DMSO_H3K27me3.normalized.bw
G477-DMSO_Input.bw
G477-K27M_H3K27me2.normalized.bw
G477-K27M_H3K27me3.normalized.bw
G477-K27M_Input.bw
G477-K27R_H3K27me2.normalized.bw
G477-K27R_H3K27me3.normalized.bw
G477-K27R_Input.bw
G477-UNC1999_H3K27me2.normalized.bw
G477-UNC1999_H3K27me3.normalized.bw
G477-UNC1999_Input.bw
G477-WT1_H3K27me3.bw
G477-WT1_Input.bw
G477-WT2_Input.bw
G477-WT2_RING1B.bw
G477-WT2_SUZ12.bw
H1_H3K27me3.normalized.bw
H1_Input.bw
H3.3K27M+PDGFRA_HA.bw
H3.3K27M_HA.bw
H3.3WT+PDGFRA_HA.bw
H3.3WT_HA.bw
HEK293T-K27M1_H3K27me3.normalized.bw
HEK293T-K27M1_Input.bw
HEK293T-K27M2_Input.bw
HEK293T-K27M2_SUZ12.bw
HEK293T-WT_H3K27me3.normalized.bw
HEK293T-WT_Input.bw
HSJ019-K27M_H3K27me2.normalized.bw
HSJ019-K27M_H3K27me3.normalized.bw
HSJ019-K27M_Input.bw
HSJ019-Tumor_H3K27me3.normalized.bw
HSJ019-Tumor_Input.bw
pcGBM2-WT1_H3K27me3.normalized.bw

pcGBM2-WT1_Input.bw
pcGBM2-WT2_H3K27me3.bw
pcGBM2-WT2_Input.bw
pcGBM2-WT3_Input.bw
pcGBM2-WT3_RING1B.bw
pcGBM2-WT3_SUZ12.bw
pcGBM2-WT4_H3K27me2.normalized.bw
pcGBM2-WT4_Input.bw
1840-Tumor_H3K27me3.bw
2230-Tumor_H3K27me3.bw
BTTB434-Tumor_H3K27me3.bw
JN57-Tumor_H3K27me3.bw
PH1157-Tumor_H3K27me3.bw
PS113293-Tumor_H3K27me3.bw
HSJ019-Tumor_H3K27me3.bw
1840-Tumor_H3K27me3_1.fastq.gz
1840-Tumor_H3K27me3_2.fastq.gz
2230-Tumor_H3K27me3_1.fastq.gz
2230-Tumor_H3K27me3_2.fastq.gz
BT245-EZH2-Y641N_H3K27me2.bam
BT245-EZH2-Y641N_H3K27me3.bam
BT245-EZH2-Y641N_Input.bam
BT245-EZH2-wt_H3K27me2.bam
BT245-EZH2-wt_H3K27me3.bam
BT245-EZH2-wt_Input.bam
BT245-K27M1_H3K27me2.bam
BT245-K27M1_H3K27me3.bam
BT245-K27M1_Input.bam
BT245-K27M2_H3K27me2.bam
BT245-K27M2_H3K27me3.bam
BT245-K27M2_Input.bam
BT245-K27M2_SUZ12.bam
BT245-K27M3_H3.3.bam
BT245-K27M3_input_1.bam
BT245-K27M3_input_2.bam
BT245-K27M4_Input.bam
BT245-K27M4_K27M.bam
BT245-K27M5_Input.bam
BT245-K27M5_RING1B.bam
BT245-K27M5_SUZ12.bam
BT245-KO1-r2_H3K27me2.bam
BT245-KO1-r2_Input.bam
BT245-KO1_H3K27me3.bam
BT245-KO1_Input.bam
BT245-KO2-r2_H3K27me2.bam
BT245-KO2-r2_Input.bam
BT245-KO2-r2_SUZ12.bam
BT245-KO2_H3K27me3.bam
BT245-KO2_Input.bam
BT245-UNC1999_H3K27me2.bam
BT245-UNC1999_H3K27me3.bam
BT245-UNC1999_Input.bam
BT416-K27M_H3K27me3_1.bam
BT416-K27M_H3K27me3_2.bam
BT416-K27M_Input_1.bam
BT416-K27M_Input_2.bam
BTTB434-Tumor_H3K27me3.fastq.gz
DIPGVI-K27M1_H3K27me3.bam
DIPGVI-K27M1_Input_1.bam
DIPGVI-K27M1_Input_2.bam
DIPGVI-K27M2_Input.bam
DIPGVI-K27M2_RING1B.bam
DIPGVI-K27M2_SUZ12.bam
DIPGXIII-K27M-DMSO_H3K27me3.bam
DIPGXIII-K27M-DMSO_Input.bam
DIPGXIII-K27M-DMSO_SUZ12.bam
DIPGXIII-K27M1_H3K27me2.bam
DIPGXIII-K27M1_H3K27me3.bam
DIPGXIII-K27M1_Input.bam
DIPGXIII-K27M1_K27M.bam
DIPGXIII-K27M2_H3.3.bam
DIPGXIII-K27M2_Input_1.bam
DIPGXIII-K27M2_Input_2.bam
DIPGXIII-K27M3_Input.bam
DIPGXIII-K27M3_RING1B.bam

DIPGXIII-K27M3_SUZ12.bam
DIPGXIII-KO1_H3K27me2.bam
DIPGXIII-KO1_H3K27me3.bam
DIPGXIII-KO1_Input.bam
DIPGXIII-KO1_SUZ12.bam
DIPGXIII-KO2_H3K27me2.bam
DIPGXIII-KO2_H3K27me3.bam
DIPGXIII-KO2_Input.bam
DIPGXIII-UNC1999_H3K27me3.bam
DIPGXIII-UNC1999_Input.bam
G477-DMSO2_H3K27me2.bam
G477-DMSO2_Input_1.bam
G477-DMSO2_Input_2.bam
G477-DMSO3_H3K27me2.bam
G477-DMSO3_Input.bam
G477-DMSO_H3K27me3.bam
G477-DMSO_Input.bam
G477-K27M_H3K27me2.bam
G477-K27M_H3K27me3.bam
G477-K27M_Input.bam
G477-K27R_H3K27me2.bam
G477-K27R_H3K27me3.bam
G477-K27R_Input.bam
G477-UNC1999_H3K27me2.bam
G477-UNC1999_H3K27me3.bam
G477-UNC1999_Input.bam
G477-WT1_H3K27me3_1.bam
G477-WT1_H3K27me3_2.bam
G477-WT1_Input_1.bam
G477-WT1_Input_2.bam
G477-WT2_Input.bam
G477-WT2_RING1B.bam
G477-WT2_SUZ12.bam
H1_H3K27me3.bam
H1_Input.bam
H3.3K27M+PDGFRA_HA_1.bam
H3.3K27M+PDGFRA_HA_2.bam
H3.3K27M+PDGFRA_HA_3.bam
H3.3K27M_HA_1.bam
H3.3K27M_HA_2.bam
H3.3K27M_HA_3.bam
H3.3WT+PDGFRA_HA_1.bam
H3.3WT+PDGFRA_HA_2.bam
H3.3WT+PDGFRA_HA_3.bam
H3.3WT_HA_1.bam
H3.3WT_HA_2.bam
H3.3WT_HA_3.bam
HEK293T-K27M1_H3K27me3.bam
HEK293T-K27M1_Input.bam
HEK293T-K27M2_Input.bam
HEK293T-K27M2_SUZ12.bam
HEK293T-WT_H3K27me3.bam
HEK293T-WT_Input.bam
HSJ019-K27M_H3K27me2.bam
HSJ019-K27M_H3K27me3.bam
HSJ019-K27M_Input_1.bam
HSJ019-K27M_Input_2.bam
HSJ019-Tumor_H3K27me3.bam
HSJ019-Tumor_Input.bam
JN57-Tumor_H3K27me3.bam
PH1157-Tumor_H3K27me3.fastq.gz
PS113293-Tumor_H3K27me3.fastq.gz
pcGBM2-WT1_H3K27me3.bam
pcGBM2-WT1_Input.bam
pcGBM2-WT3_Input.bam
pcGBM2-WT3_RING1B.bam
pcGBM2-WT3_SUZ12.bam
pcGBM2-WT4_H3K27me2.bam
pcGBM2-WT4_Input_1.bam
pcGBM2-WT4_Input_2.bam
pcGBM2_WT2_H3K27me3_1.bam
pcGBM2_WT2_H3K27me3_2.bam
pcGBM2_WT2_Input_1.bam
pcGBM2_WT2_Input_2.bam
BT245-K27M-r1.bam

BT245-K27M-r2_1.bam
BT245-K27M-r2_2.bam
BT245-K27M-r3_1.bam
BT245-K27M-r3_2.bam
BT245-K27M-r4.bam
BT245-K27M-r5.bam
BT245-K27M-r6.bam
BT245-KO-r1.bam
BT245-KO-r2.bam
BT245-KO-r3.bam
BT245-KO-r4.bam
BT245-KO-r5_1.bam
BT245-KO-r5_2.bam
BT245-KO-r6_1.bam
BT245-KO-r6_2.bam
DIPGXIII-K27M-r1.bam
DIPGXIII-K27M-r2.bam
DIPGXIII-KO-r1.bam
DIPGXIII-KO-r2.bam
G477-K27M-r1.bam
G477-K27M-r2.bam
G477-K27M-r3.bam
G477-K27R-r1.bam
G477-K27R-r2.bam
G477-K27R-r3.bam
HEK293T-K27M-r1.bam
HEK293T-K27M-r2.bam
HEK293T-K27M-r3.bam
HEK293T-WT-r1.bam
HEK293T-WT-r2.bam
HEK293T-WT-r3.bam
BT245-K27M_WGBS.tdf
DIPGXIII-K27M_WGBS.tdf
G477-WT_WGBS.tdf
BT245-K27M_WGBS_1.bam
BT245-K27M_WGBS_2.bam
BT245-K27M_WGBS_3.bam
DIPGXIII-K27M_WGBS_R1.fastq.gz
DIPGXIII-K27M_WGBS_R2.fastq.gz
G477-WT_WGBS_1.bam
G477-WT_WGBS_2.bam
G477-WT_WGBS_3.bam

Genome browser session
(e.g. UCSC)

Not applicable - data visualization using IGV.

Methodology

Replicates

No technical replicates. All replicates are biological.

Sequencing depth

ChIP-seq: 50 bp, single-end

BT245-EZH2-wt_H3K27me3.normalized, total number of reads: 53477100
BT245-EZH2-wt_Input, total number of reads: 33437727
BT245-EZH2-Y641N_H3K27me3.normalized, total number of reads: 47800923
BT245-EZH2-Y641N_Input, total number of reads: 57761508
BT245-K27M1_H3K27me3.normalized, total number of reads: 83947640
BT245-K27M1_Input, total number of reads: 20217214
BT245-K27M2_H3K27me3.normalized, total number of reads: 50886206
BT245-K27M2_Input, total number of reads: 32459188
BT245-K27M2_SUZ12, total number of reads: 71561214
BT245-K27M3_H3.3, total number of reads: 16069492
BT245-K27M3_input, total number of reads: 63740926
BT245-K27M4_Input, total number of reads: 35937894
BT245-K27M4_K27M, total number of reads: 55062772
BT245-K27M5_Input, total number of reads: 35978164
BT245-K27M5_RING1B, total number of reads: 24265840
BT245-K27M5_SUZ12, total number of reads: 24154322
BT245-KO1_H3K27me3.normalized, total number of reads: 38412661
BT245-KO1_Input, total number of reads: 45643720
BT245-KO2_H3K27me3.normalized, total number of reads: 50533762
BT245-KO2_Input, total number of reads: 36722196
BT245-KO2-r2_Input, total number of reads: 56241627
BT245-KO2-r2_SUZ12, total number of reads: 71463402
BT416-K27M_H3K27me3, total number of reads: 64509186

BT416-K27M_Input, total number of reads: 32171696
DIPGVI-K27M1_H3K27me3, total number of reads: 6083424
DIPGVI-K27M1_Input, total number of reads: 30101993
DIPGVI-K27M2_Input, total number of reads: 19285355
DIPGVI-K27M2_RING1B, total number of reads: 25258990
DIPGVI-K27M2_SUZ12, total number of reads: 27255209
DIPGXIII-K27M1_H3K27me3.normalized, total number of reads: 66094572
DIPGXIII-K27M1_Input, total number of reads: 38249283
DIPGXIII-K27M1_K27M, total number of reads: 68670294
DIPGXIII-K27M2_H3.3, total number of reads: 14992388
DIPGXIII-K27M2_Input, total number of reads: 67709978
DIPGXIII-K27M3_Input, total number of reads: 20846980
DIPGXIII-K27M3_RING1B, total number of reads: 28096566
DIPGXIII-K27M3_SUZ12, total number of reads: 23138764
DIPGXIII-K27M-DMSO_H3K27me3.normalized, total number of reads: 66874353
DIPGXIII-K27M-DMSO_Input, total number of reads: 39166389
DIPGXIII-K27M-DMSO_SUZ12, total number of reads: 46254161
DIPGXIII-KO1_H3K27me3.normalized, total number of reads: 50587171
DIPGXIII-KO1_Input, total number of reads: 37606763
DIPGXIII-KO1_SUZ12, total number of reads: 56385240
DIPGXIII-KO2_H3K27me3.normalized, total number of reads: 54113261
DIPGXIII-KO2_Input, total number of reads: 36719138
DIPGXIII-UNC1999_H3K27me3.normalized, total number of reads: 67782649
DIPGXIII-UNC1999_Input, total number of reads: 57716311
G477-DMSO_H3K27me3.normalized, total number of reads: 50461231
G477-DMSO_Input, total number of reads: 35328596
G477-K27M_H3K27me3.normalized, total number of reads: 35793870
G477-K27M_Input, total number of reads: 35583116
G477-K27R_H3K27me3.normalized, total number of reads: 41023809
G477-K27R_Input, total number of reads: 33834424
G477-UNC1999_H3K27me3.normalized, total number of reads: 87800066
G477-UNC1999_Input, total number of reads: 34254808
G477-WT1_H3K27me3, total number of reads: 65733551
G477-WT1_Input, total number of reads: 44562808
G477-WT2_Input, total number of reads: 21807696
G477-WT2_RING1B, total number of reads: 27252617
G477-WT2_SUZ12, total number of reads: 23590152
H1_H3K27me3.normalized, total number of reads: 36656450
H1_Input, total number of reads: 42536700
H3.3K27M_HA, total number of reads: 73030762
H3.3K27M+PDGFRA_HA, total number of reads: 66335173
H3.3WT_HA, total number of reads: 58451552
H3.3WT+PDGFRA_HA, total number of reads: 64778504
HEK293T-K27M1_H3K27me3.normalized, total number of reads: 67123256
HEK293T-K27M1_Input, total number of reads: 36172165
HEK293T-K27M2_Input, total number of reads: 36915717
HEK293T-K27M2_SUZ12, total number of reads: 28801561
HEK293T-WT_H3K27me3.normalized, total number of reads: 62591924
HEK293T-WT_Input, total number of reads: 50569699
HSJ019-K27M_H3K27me3.normalized, total number of reads: 53624858
HSJ019-K27M_Input, total number of reads: 16569243
HSJ019-Tumor_H3K27me3.normalized, total number of reads: 72014652
HSJ019-Tumor_Input, total number of reads: 42428099
pcGBM2_WT2_H3K27me3, total number of reads: 62908105
pcGBM2_WT2_Input, total number of reads: 34849258
pcGBM2-WT1_H3K27me3.normalized, total number of reads: 57543175
pcGBM2-WT1_Input, total number of reads: 47687618
pcGBM2-WT3_Input, total number of reads: 35978164
pcGBM2-WT3_RING1B, total number of reads: 25258990
pcGBM2-WT3_SUZ12, total number of reads: 27483071
BT245-K27M1_H3K27me2.normalized, total number of reads: 51217850
BT245-EZH2-wt_H3K27me2.normalized, total number of reads: 84318751
BT245-EZH2-Y641N_H3K27me2.normalized, total number of reads: 55979653
BT245-KO1-r2_H3K27me2.normalized, total number of reads: 70607562
BT245-KO2-r2_H3K27me2.normalized, total number of reads: 82735810
BT245-K27M2_H3K27me2.normalized, total number of reads: 73456978
BT245-UNC1999_H3K27me2.normalized, total number of reads: 75462538
DIPGXIII-K27M1_H3K27me2.normalized, total number of reads: 65698345
DIPGXIII-KO2_H3K27me2.normalized, total number of reads: 72997772
DIPGXIII-KO1_H3K27me2.normalized, total number of reads: 66243495
G477-DMSO2_H3K27me2.normalized, total number of reads: 59660311
G477-DMSO3_H3K27me2.normalized, total number of reads: 86526376
G477-K27M_H3K27me2.normalized, total number of reads: 61231418
G477-K27R_H3K27me2.normalized, total number of reads: 67078915
G477-UNC1999_H3K27me2.normalized, total number of reads: 97507665

pcGBM2-WT4_H3K27me2.normalized, total number of reads: 66131872
HSJ019-K27M_H3K27me2.normalized, total number of reads: 54361222
BT245-UNC1999_H3K27me3.normalized, total number of reads: 71983596
BT245-KO1-r2_Input, total number of reads: 64210157
BT245-UNC1999_Input, total number of reads: 33670928
pcGBM2-WT4_Input, total number of reads: 71974790
G477-DMSO2_Input, total number of reads: 70209383
G477-DMSO3_Input, total number of reads: 54303916
1840-Tumor_H3K27me3, total number of reads: 36844329
2230-Tumor_H3K27me3, total number of reads: 56427318
BTTB434-Tumor_H3K27me3, total number of reads: 56364363
HSJ019-Tumor_H3K27me3, total number of reads: 68296718
JN57-Tumor_H3K27me3, total number of reads: 18593382
PH1157-Tumor_H3K27me3, total number of reads: 36931977
PS113293-Tumor_H3K27me3, total number of reads: 32322558

RNA-seq: 125 bp, paired-end

BBT245-K27M-r1, total number of reads: 95403926
BT245-K27M-r2_1, total number of reads: 95618192
BT245-K27M-r2_2, total number of reads: 95618192
BT245-K27M-r3_1, total number of reads: 92044601
BT245-K27M-r3_2, total number of reads: 92044601
BT245-K27M-r4, total number of reads: 119641772
BT245-K27M-r5, total number of reads: 129288012
BT245-K27M-r6, total number of reads: 121301782
BT245-KO-r1, total number of reads: 98915500
BT245-KO-r2, total number of reads: 103340480
BT245-KO-r3, total number of reads: 121127276
BT245-KO-r4, total number of reads: 106948788
BT245-KO-r5_1, total number of reads: 77222297
BT245-KO-r5_2, total number of reads: 77222297
BT245-KO-r6_1, total number of reads: 88676481
BT245-KO-r6_2, total number of reads: 88676481
DIPGXIII-K27M-r1, total number of reads: 129746094
DIPGXIII-K27M-r2, total number of reads: 107791858
DIPGXIII-KO-r1, total number of reads: 127626360
DIPGXIII-KO-r2, total number of reads: 113809530
G477-K27M-r1, total number of reads: 163454018
G477-K27M-r2, total number of reads: 136875760
G477-K27M-r3, total number of reads: 159286888
G477-K27R-r1, total number of reads: 129569616
G477-K27R-r2, total number of reads: 133246688
G477-K27R-r3, total number of reads: 148437814
HEK293T-WT-r1, total number of reads: 145303584
HEK293T-WT-r2, total number of reads: 152121560
HEK293T-WT-r3, total number of reads: 146848026
HEK293T-K27M-r1, total number of reads: 145353674
HEK293T-K27M-r2, total number of reads: 145916372
HEK293T-K27M-r3, total number of reads: 146464374

WGBS: 150 bp, paired-end

BT245-K27M_WGBS, total number of reads: 530199310
DIPGXIII-K27M_WGBS, total number of reads: 952732450
G477-WT_WGBS, total number of reads: 1018860094

Antibodies

For ChIP, the following primary antibodies were used: anti-H3K27me3 (Cell Signaling Tech, 9733), anti-H3K27me3 (Active Motif, 61017), anti-H3K27me2 (Cell Signaling Tech, 9728), anti-SUZ12 (Cell Signaling Tech, 3737), anti-RING1B (Active Motif, 39663), anti-H3.3 (Millipore, 09-838), anti-H3K27M (Millipore, ABE419), anti-HA (Cell Signaling Tech, 3724).

Peak calling parameters

Peak-calling was done using MACS2 version 2.1.1 with default parameters.

Data quality

ChIP-sequencing experiments were assessed for their percent of mapped reads (to hg19 and dm6, when applicable) to ensure proper coverage. Reads with poor mapping quality were discarded from further analysis. Antibody pulldown efficacy was visually assessed by looking at tracks and comparing with spiked-in distribution of marks, when applicable.

For DNA methylation, bi-sulfite conversion was confirmed by lambda-phage spike in and methylation levels of cytosines were considered. Cytosines with less than 5x coverage were discarded from further analysis.

Software

ChIP-seq raw reads were aligned to human (hg19) or mouse (mm10) and drosophila (dm6) genome build using BWA version 0.7.17 with default parameters. Read counting in bins was done using bedtools version 2.22.1. ChIP-sequencing coverage tracks were visualized using IGV 2.3 software. Peak-calling was done using MACS2 version 2.1.1. The SUZ12 peak-centered H3K27me3 enrichment plots and PMD plot were generated using ngs.plot.r package. The aggregate plots of SUZ12 and

H3K27me3 were generated using deepTools v3.1.0. Heat map plots for comparing H3K27me3, SUZ12, RING1B, and DNA methylation were generated using ChAsE v.1.0.11 software. Custom R scripts (available upon request) were used to generate the top 1% bin scores, the plots of H3K27me3 variations, the correlation plots of K27M-K27me2 and WT-K27me3 and the Decile plots.

RNA-seq raw reads were aligned to human genome build (UCSC hg19) using STAR version 2.5.3a. The mapped reads for genes were counted using featureCounts program (version 1.5.3). Gene set enrichment analysis was performed using the GSEA tool from Broad Institute.

WGBS raw reads were aligned to human genome build (UCSC hg19) using BWA (version 0.6.1). Samtools (version 0.1.18) in mpileup mode was applied to call methylation of individual CpGs.
